# Radiation‐induced C‐reactive protein triggers apoptosis of vascular smooth muscle cells through ROS interfering with the STAT3/Ref‐1 complex

**DOI:** 10.1111/jcmm.17233

**Published:** 2022-02-17

**Authors:** Je‐Won Ryu, In‐Hye Jung, Eun‐Young Park, Kang‐Hyun Kim, Kyunggon Kim, Jeonghun Yeom, Jinhong Jung, Sang‐wook Lee

**Affiliations:** ^1^ Department of Convergence Research Center, Asan Institute for Life Sciences Asan Medical Center Seoul Republic of Korea; ^2^ Department of Radiation Oncology Gang Neung Asan Medical Center Ganneung‐si Republic of Korea; ^3^ Department of Radiation Oncology Asan Medical Center University of Ulsan College of Medicine Seoul Republic of Korea

**Keywords:** atherosclerosis, C‐reactive protein, phenotype switching, radiotherapy, Redox factor‐1, vascular smooth muscle cells

## Abstract

Damage to normal tissue can occur over a long period after cancer radiotherapy. Free radical by radiation can initiate or accelerate chronic inflammation, which can lead to atherosclerosis. However, the underlying mechanisms remain unclear. Vascular smooth muscle cells (VSMCs) proliferate in response to JAK/STAT3 signalling. C‐reactive protein (CRP) can induce VSMCs apoptosis via triggering NADPH oxidase (NOX). Apoptotic VSMCs promote instability and inflammation of atherosclerotic lesions. Herein, we identified a VSMCs that switched from proliferation to apoptosis through was enhanced by radiation‐induced CRP. NOX inhibition using lentiviral sh‐p22^phox^ prevented apoptosis upon radiation‐induced CRP. CRP overexpression reduced the amount of STAT3/Ref‐1 complex, decreased JAK/STAT phosphorylation and formed a new complex of Ref‐1/CRP in VSMC. Apoptosis of VSMCs was further increased by CRP co‐overexpressed with Ref‐1. Functional inhibition of NOX or p53 also prevented apoptotic activity of the CRP‐Ref‐1 complex. Immunofluorescence showed co‐localization of CRP, Ref‐1 and p53 with α‐actin‐positive VSMC in human atherosclerotic plaques. In conclusion, radiation‐induced CRP increased the VSMCs apoptosis through Ref‐1, which dissociated the STAT3/Ref‐1 complex, interfered with JAK/STAT3 activity, and interacted with CRP‐Ref‐1, thus resulting in transcription‐independent cell death via p53. Targeting CRP as a vascular side effect of radiotherapy could be exploited to improve curability.

## 1 INTRODUCTION

Radiotherapy is prescribed for many malignancies, including over 50% of solid cancer patients, resulting in high survival rates for patients with early head and neck cancer or breast cancer.^1^, ^2^, ^3^ However, radiotherapy can cause many years later chronic inflammatory diseases affecting the vascular system, including stroke, myocardial infarction, acute artery rupture and unstable atherosclerotic plaques.^4^, ^5^ In chronic inflammation, vascular smooth muscle cells (VSMCs) undergo a phenotypic switch that involves cell proliferation, migration, death and senescence in blood vessel walls, which can proceed over several decades.^6^ Furthermore, VSMCs‐specific loss can promote a variety of inflammatory responses in blood vessels, including increased macrophage recruitment. A phenotypic switch of VSMCs from proliferation to apoptosis or cell senescence can result in advanced atherosclerosis or vascular damage, notably through plaque instability, leading to a critical condition called acute coronary syndrome.^6^ The fibrous cap weakened by VSMCs loss is also a major factor in the ruptured‐ or vulnerable plaques formation.^5^, ^6^, ^7^, ^8^ Providing a mechanistic understanding of the molecular mediators able to modify this phenotype is important to prevent side effects and improve radiotherapy.

Because the marker of inflammation C‐reactive protein (CRP) is sensitive to radiation, serum CRP levels are measured in biodosimetry following radiation accidents.^9^ Blood CRP values of 30 rhesus monkeys were increased in a dose‐ and time‐dependent manner upon exposure to 1–8.5 Gy of 60 Co γ‐rays.^10^ The level of CRP reflects the time course and inflammatory severity of radiotherapy and can predict the outcomes of cancer patients.^11^ A high level of CRP is also a strong predictor of atherosclerosis and is closely associated with plaque instability histologically and clinically.^12^, ^13^, ^14^ CRP is expressed in apoptotic artery VSMCs,^15^ where it contributes to plaque rupture through the upregulation of NADPH oxidase (NOX).^16^ In a previous study, we found that CRP can induce apoptosis of VSMC via NOX4 activation.^17^ However, the pathophysiological mechanisms underlying the involvement of elevated CRP in the development of vulnerable plaques and the loss of VSMC remain unclear.

A ubiquitous human AP‐endonuclease/Redox factor‐1 (APE/Ref‐1) plays multifunctional roles including repairing damaged DNA via the base excision repair pathway and regulating as a co‐factor different transcriptional factors in controlling different cellular processes such as apoptosis, proliferation and differentiation.^18^, ^19^ Ref‐1 was shown to inhibit excessive ROS production related to NOX and NF‐κB activation.^18^ Additionally, Ref‐1 was found to be implicated in cardiovascular diseases.^19^, ^20^, ^21^ Ref‐1 also contributes to vessel wall changes from the Go/G1 to S phase in VSMC.^22^ Ref‐1 can bind to the acetylated NH2 terminus of STAT3 for a stable chromatin association, while Ref‐1 can lead to transcriptional elongation of STAT3 via complex formation of STAT3/Ref‐1 in the nucleus.^23^, ^24^ Proteins such as CRP, Ref‐1 and STAT3 have been reported in many studies to regulate cell death or proliferation,^15^, ^17^, ^24^, ^25^ but their interactions remain unclear.

Vascular smooth muscle cells loss by apoptosis is a critical factor in driving vulnerable plaque formation by affecting vessel remodelling, inflammation and coagulation.^7^ However, in numerous studies, VSMC proliferation has also been implicated in atherosclerosis and restenosis.^6^, ^26^ Blocking VSMC proliferation has been proposed as a therapeutic strategy by many studies.^26^, ^27^ Consequently, both the proliferation and apoptosis of VSMCs are associated with atherosclerosis exacerbation. So far, the molecular mechanisms responsible for vessel wall damage in the chronic inflammation that follows radiation therapy have not been elucidated.

The purpose of this study was to determine whether changes in Ref‐1 activity caused by CRP accumulation could regulate the survival and death of VSMCs during vessel damage.

## 2 MATERIALS AND METHODS

### 2.1 Cell culture

Human aortic VSMCs (PCS‐100‐012; American Type Culture Collection, Manassas, VA, USA) were grown to confluence in medium (PCS‐100‐030 and PCS‐100‐042; American Type Culture Collection). Rat VSMCs (A10; American Type Culture Collection) were cultured in DMEM medium with 1500 mg/L sodium bicarbonate (Gibco BRL) in a 5% CO_2_/37°C incubator.

### 2.2 Plasmids

Human CRP cDNA (GeneBank accession No. NM_000567) was PCR amplified using a pair of specific primers, 5′‐TGAATTCAGGCCCTTGTATC‐3′ (sense) and 5′‐TCCCAGCATAGTTAACGAGC‐3′ (antisense) and human Ref‐1 (GeneBank accession No. NM_001641) was PCR amplified by specific primers, 5′‐AAGCTTGAGTCAGGACCCTT‐3′ (sense) and 5′‐AAGGAATGGTAGTTGAGGGG‐3′ (antisense), for cloning. The complete nucleotide sequences were cloned into the pcDNA3.1 expression vector (CRP‐pcDNA3.1) (Invitrogen) and were sub‐cloned into the pEF1α‐Myc, pDsRed1‐N1 and pEGFP‐N2 vectors (Clontech Laboratories, Inc.).

### 2.3 Generation of cell lines with stable knockdown using a lentiviral shRNA system

pLKO.1‐puro‐human CYBA (p22^phox^; TRCN0000064581) and U6‐EGFP (SHC005) control lentiviral vectors were transformed primary embryonic kidney cells (293FT; Invitrogen) then used for packaging lentiviruses (cotransfection of pRSV‐Rev, pMDLg/pRRE and pMD2.G; 3rd generation transfer plasmids, Addgene) including the sh p22^phox^ lentiviral vector, as described previously.^28^

### 2.4 Quantification of intracellular ROS

ROS was detected using 5 μM H**_2_** DCFDA and 5 μM MitoSOX™ (mitochondrial ROS indicator; Molecular Probes, Invitrogen) as previously described.^29^ The amount of ROS was analysed by flow cytometry (CELLQUEST software; BD Biosciences).

### 2.5 Analysis of apoptotic genes mRNA expression

Specific sequences for PCR were amplified using the primers and number of cycles (94°C for 30 s, 58°C for 40 s and 72°C for 1 min) listed in Table S1.

### 2.6 Ionized radiation (IR)

The monolayers on flasks were irradiated with 6MV photons produced by a medical linear accelerator (Varian Clinac 21EX; Varian). Luminescence dosimeters (nanoDOT; Landauer) were used to measure the dose delivered by the Varian linear accelerator. Calibration of the dosimeters was performed using a MicroStar InLight reader (Landauer), and the error was <1% compared with the planned values for the cell lines (hVSMCs and A10 cells) at 2, 4 and 8 Gy.^30^

### 2.7 Staining of Annexin V and propidium iodide (PI)

IR‐exposed or IR‐overexpressed CRP in A10 cells (1 × 10^5^ cells per four‐well cultured plate) were measured using the Annexin V‐FITC apoptosis detection kit (CELLQUEST software; BD Pharmingen) after 16 or 24 h. Positive staining was screened using a confocal microscopy system (LSM710 Carl Zeiss GmbH).

### 2.8 Caspase‐3 activity assay

Caspase‐3 activity was measured using a Vybrant^®^ FAM caspase‐3 and caspase‐7 assay kit (V35118 of Molecular Probes Invitrogen, Fisher Scientific). Briefly, IR‐exposed VSMCs and cDNA‐transfected A10 cells (CRP or Ref‐1) were pretreated with 1 μM diphenylene iodonium (DPI) or 500 μM N‐acetyl‐I‐cysteine (NAC) for 24 h. Caspase‐3 activation can be analysed by flow cytometry (CELLQUEST software; BD Pharmingen) using 488 nm excitation.

### 2.9 TUNEL assay

Apoptotic VSMCs were detected by TUNEL staining using the TdT‐FagELTM kit (Oncogene Research Products) for 16 or 48 h and analysed by confocal microscopy (TCS‐SP2 system; Leica Microsystems).

### 2.10 Preparation of cytoplasmic and nuclear extracts

Nuclear extracts were prepared as described previously.^31^ Briefly, Nuclei were collected by centrifugation at 2,860 *g*  at 4°C for 10 min. The supernatant was considered the cytoplasmic fraction. Cytoplasmic extracts were subsequently centrifuged at 11,460 *g* for 20 min at 4°C.

### 2.11 Isolation of mitochondria

Mitochondrial fractions were isolated from 2 × 10^7^ VSMCs using the Mitochondria Isolation Kit (Thermo Scientific Pierce Biotechnology) according to the manufacturer's instructions.

### 2.12 Immunoblotting (IB) assay

Western blot was performed using primary antibodies against the proteins of interest as previously described.^17^ The antibodies are listed in Table S2. The protein amount was quantified by scanning photo‐densitometry and its quantitation software (MULTI‐IMAGE & Bio‐Rad Laboratories Inc.).

### 2.13 Immunoprecipitation (IP)

The VSMCs were plated on 100 mm dishes and transfected with, CRP‐ or REF‐pcDNA3.1, pEF1**α**‐myc‐hCRP or pEF1**α**‐myc‐hRef‐1. The immunoprecipitants were subjected to Western blotting using the indicated antibodies.^17^

### 2.14 Immunofluorescence (IF)

Fluorescence‐tagged secondary antibodies were used to reveal the primary antibody (Table S2). Nuclei were stained with 1 mM 4′,6‐diamidino‐2‐phenylindole (DAPI). Mitochondria were stained with MitoTracker™ Red CMXRos (M7512; Thermo Fisher Scientific). Images were collected by confocal microscopy (TCS‐SP2; Leica Microsystems or LSM 710; Carl Zeiss Co., Ltd.).

### 2.15 Statistical analysis

The SPSS package was used to perform the statistical analyses. The results are shown as mean ± standard deviation (SD) of three independent experiments. Means for different groups were compared using unpaired, two‐tailed Student's *t*‐tests (***p < 0.001, **p < 0.01, *p < 0.05).

## 3 RESULTS

### 3.1 Ionizing radiation enhances ROS production, CRP and Ref‐1 expression in VSMCs

**FIGURE 1:**
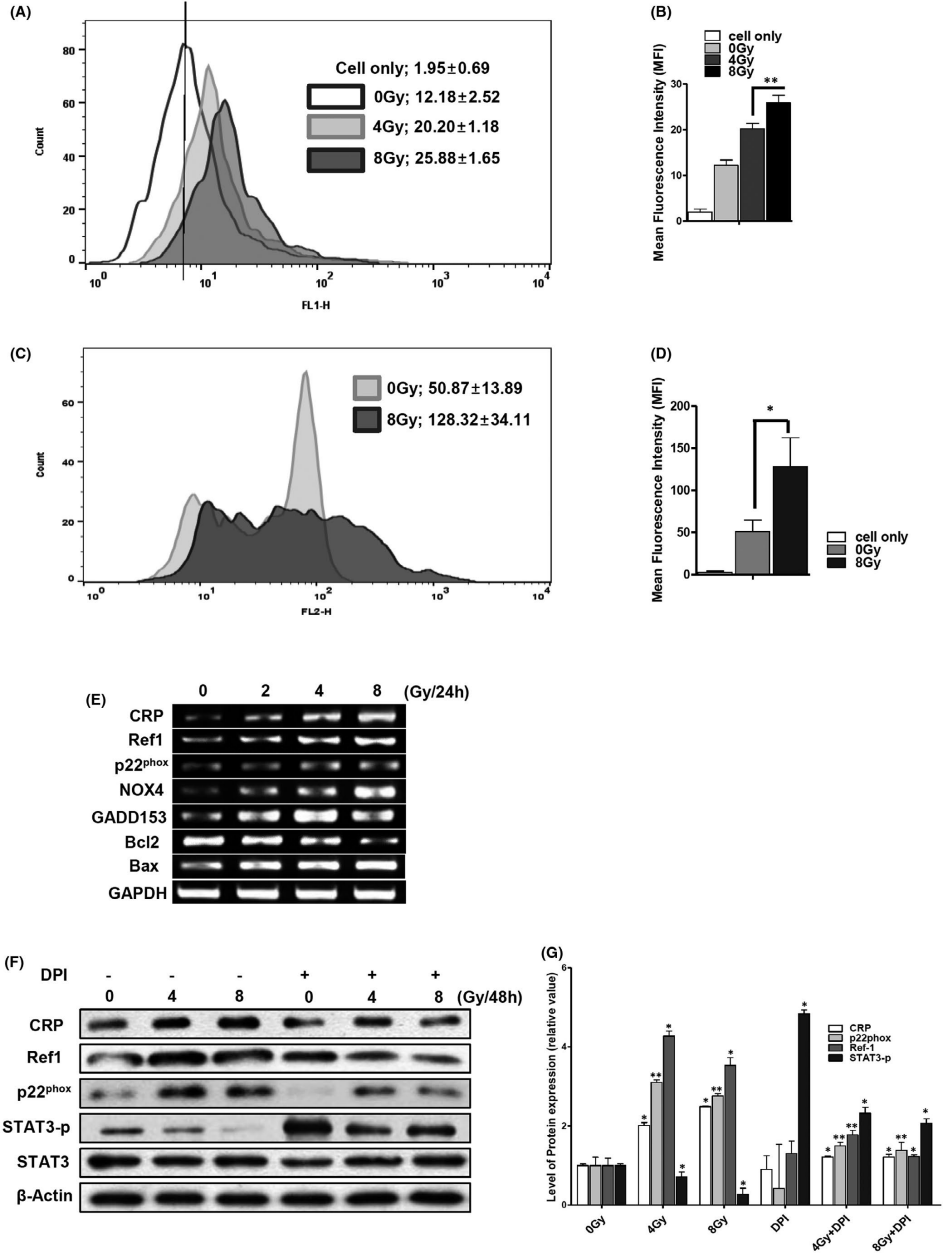
IR enhances ROS production and CRP expression in VSMCs. Intracellular ROS production and gene expression were analysed by exposing human aortic VSMCs to ionizing radiation. (A, B) Intracellular ROS of hVSMCs was analysed by flow cytometry with 5 μM H_2_DCFDA for 15 min and exciting at 488 nM (FL1: green). (C, D) Mitochondrial ROS of hVSMCs was analysed by flow cytometry with 5 μM mitoSOX™ and exited at 510 nm (FL2: red). (B, D) The results show the mean ± SD value of specific cell‐associated fluorescence intensity. The level of ROS production was analysed with *t*‐tests (**p < 0.01; *p < 0.05). (E) CRP, Ref‐1, p22^phox^, NOX4, GADD153, Bcl2, Bax and GAPDH mRNA levels were analysed by RT‐PCR. (F) hVMSCs were pretreated with/without DPI (1 µM) before IR exposure. The expression of CRP, Ref‐1, p22^phox^, STAT3 and phosphorylated STAT3 in IR‐exposed VSMCs was analysed by IB at 48 h. (G) Fold‐changes are presented in the bar graph. Data are representative of three independent experiments and were analysed using unpaired *t*‐tests (**p < 0.01; *p < 0.05)

In radiotherapy, cellular damage is caused by ionized radiation, generating a variety of ROS.^2^, ^32^ In general, ROS generation by radiation disappears momentarily. However, intracellular ROS can be continuously generated for 48 h and longer by activation of NOXs through secondary cell signalling due to radical stress in blood vessel cells.^33^ IR‐exposed VSMCs produced intracellular ROS dose‐dependently even after 48 h. Mean fluorescence intensity (MFI) of intracellular ROS in 0 G, 4 Gy and 8 Gy IR‐exposed VSMCs was 12.18 ± 2.5, 20.20 ± 1.2 and 25.88 ± 1.7 respectively (Figure 1A,B). Low‐dose cellular exposure to IR (0.1–10 Gy) can induce mitochondrial dysfunction via an accumulation of mitochondrial ROS.^32^ The 8 Gy‐exposed VSMCs produced significantly more mitochondrial ROS at 48 h. Mitochondrial ROS MFI of 0 Gy‐ and 8 Gy‐exposed VSMCs were 50.87 ± 13.89 and 128.31 ± 34.11 respectively, which were two times higher than the control (Figure 1C,D). CRP is also a biomarker of biodosimetry,^34^ as its accumulation in patients undergoing radiotherapy can reflect various chronic inflammatory diseases.^35^ In IR‐exposed VSMC, radiation dose‐dependently increased the CRP mRNA level after 24 h (Figure 1E). The expression levels of Ref‐1, which is known to regulate redox homeostasis,^20^ were further increased by radiation (Figure 1E–G). IR also dose‐dependently increased the mRNA levels of NOX4, GADD153 and Bax (Figure 1E). The expression of p22^phox^, which is a major subunit of NOXs,^36^ was additionally increased by radiation dose‐dependently (Figure 1E–G). We have previously reported that the accumulation of CRP enhances apoptotic damage of VSMCs via ROS production of NOX4.^17^ Phosphorylated STAT3, which increases VSMCs proliferation, was reduced by IR, while CRP and Ref‐1 levels were increased (Figure 1F–G). These results together suggest that IR can disrupt the homeostatic redox system by simultaneously increasing the production of ROS, together with the expression of CRP, Ref‐1 and apoptotic genes.

### 3.2 Ionizing radiation enhances apoptosis of VSMCs through ROS production by NOX

**FIGURE 2:**
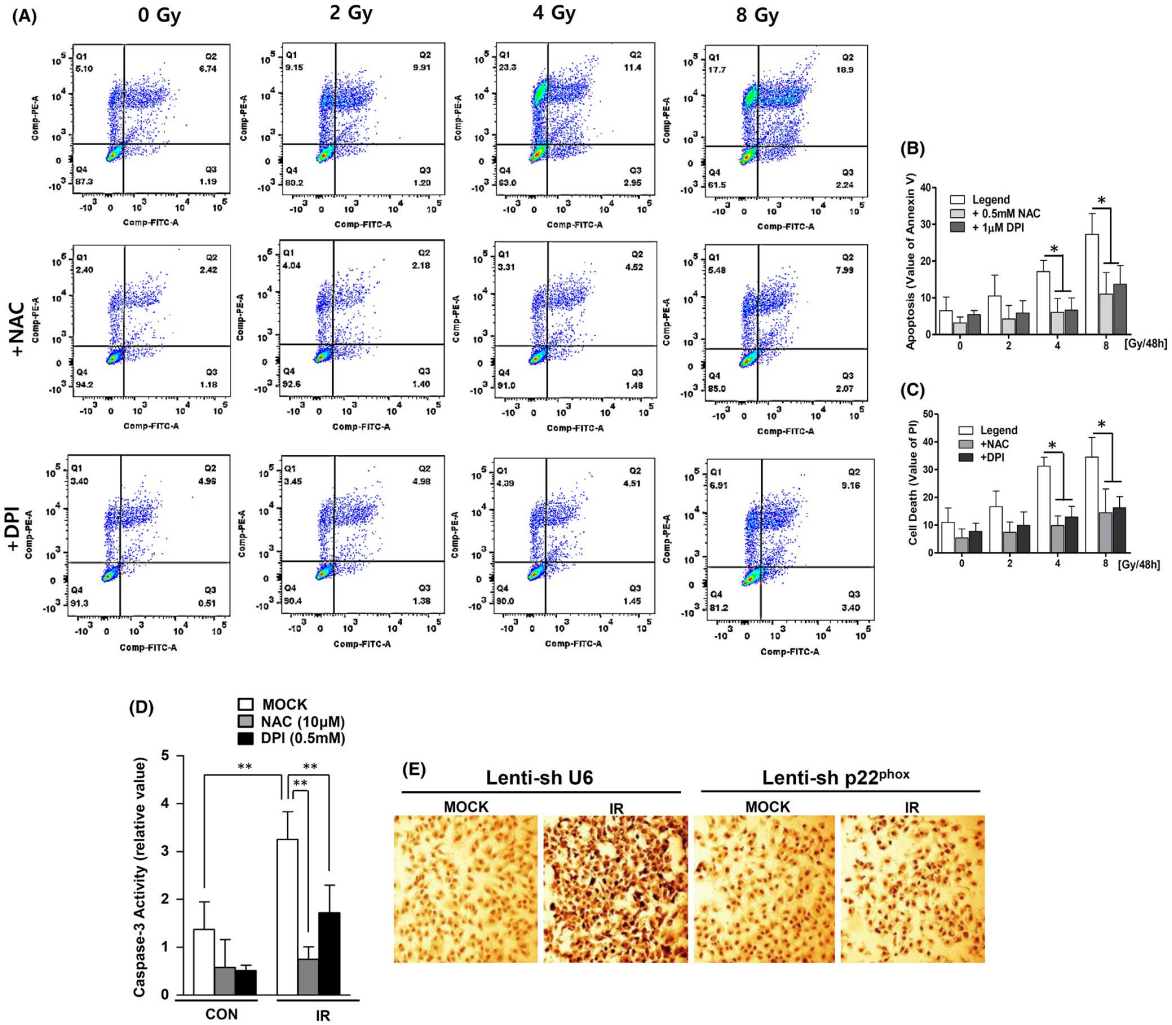
IR enhances apoptosis of VSMCs by ROS production through NOXs. (A) Apoptosis and cell death were analysed by flow cytometry using Annexin V and PI staining in IR‐exposed hVSMCs. (B, C) The mean value of the specific cell‐associated fluorescence with unpaired *t*‐tests (* *p* < 0.05). (D) Apoptosis was measured with Vybrant^®^ FAM caspase‐3 assay kits. The cells were treated with DPI (1 µM) or NAC (0.5 mM) before IR exposure. The mean ± SD value of excitation at 488 nM with unpaired *t*‐tests (** *p* < 0.01). (E) The representative images of DNA damage were analysed with TUNEL assays. Magnification, 200×. Confocal microscopic images are shown with the black colour marking the damaged DNA in the nucleus in IR‐exposed hVMSCs and p22^phox^ KD cells. The results of three independent experiments are shown

Measuring ROS production from NOXs is particularly relevant to understanding the roles of ROS in the physiology and pathophysiology of VSMCs.^37^ IR increased the apoptosis of human VSMCs dose‐dependently after 48 h (gate % values of Annexin V and PI at 0–8 Gy: control, 17.47 ± 8.85; 2 Gy, 27.21 ± 11.12; 4 Gy, 48.44 ± 3.04; 8 Gy, 61.82 ± 9.53) (Figure 2A–C). To determine whether ROS production through IR‐induced NOX enhanced VSMC apoptosis, apoptosis was measured under the same conditions after pretreatment with DPI or NAC. The apoptosis of VSMCs was found to be reduced by about 50% upon NOX‐specific inhibition of DPI (4 Gy‐exposed cells vs. DPI pretreated 4 Gy‐exposed cells: 48.44 ± 3.04 vs. 25.59 ± 7.077; 8 Gy‐exposed cells vs. DPI pretreated 8 Gy‐exposed cells: 61.82 ± 9.53 vs. 29.99 ± 9.03). Apoptosis was further attenuated following pretreatment with NAC, a ROS scavenger, in human VSMCs (4 Gy‐exposed cells vs. NAC pretreated 4 Gy‐exposed cells: decreasing 32%; 8 Gy‐exposed cells vs. NAC pretreated 8 Gy‐exposed cells: decreasing 41%) (Figure 2A–C). IR exposure also increased caspase‐3 activity more than three times (CON vs. 8 Gy: 120% ± 20% vs. 320% ± 40%). However, the IR‐induced caspase‐3 activity decreased by 47.1% following 1 μM DPI pretreatment and by 77% following 500 μM NAC pretreatment compared with the control group (Figure 2D). These results suggest that apoptosis can lead to an inhibition of caspase‐3 activity through the depletion of ROS. In VSMCs from atherosclerosis patient samples, p22^phox^ is an essential component of isotypes of NOXs.^38^ It has been shown that p22^phox^ deficiency can inhibit activation of NOXs.^17^, ^28^, ^36^, ^39^ To confirm this finding, a stable p22^phox^‐knockdown VSMC (p22^phox^ KD) was constructed (Figure S1). Confocal images showed that the IR‐induced DNA damage in VSMCs was also markedly reduced in p22^phox^ KD (Figure 2E). These results overall suggest that IR induces apoptosis by increasing ROS production of NOX in VSMCs.

### 3.3 Ionized radiation increases VSMCs apoptosis through CRP‐mediated ROS production

**FIGURE 3:**
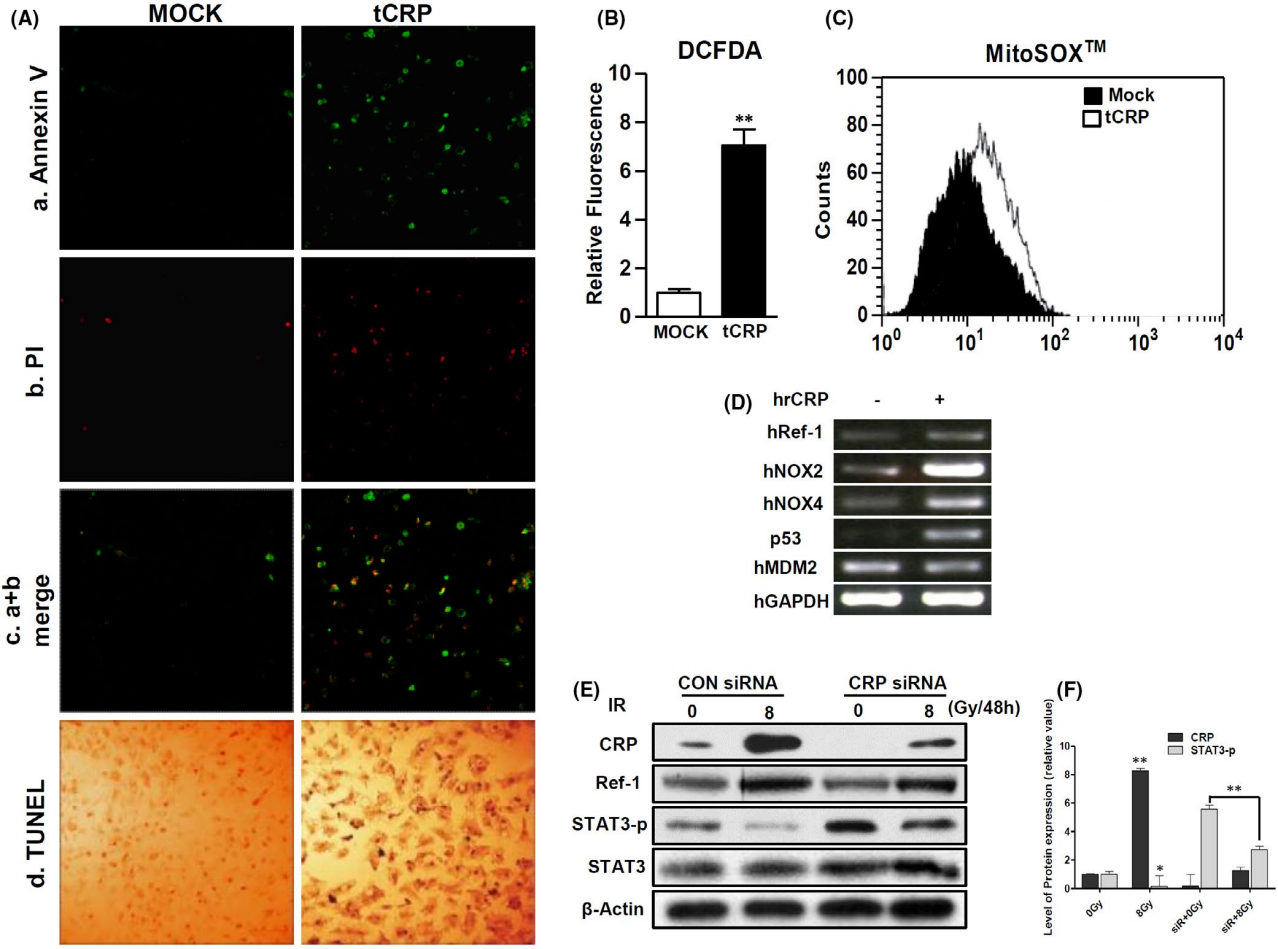
CRP‐induced ROS production increases apoptosis. (A) 1 × 10^5^ rat VSMCs (A10) were transfected with pcDNA3.1 (tMOCK) or pcDNA3.1‐human CRP cDNA (tCRP) expression vector for 24 h. CRP overexpressing cells were obtained using Annexin V‐FITC (green in Panel a) and propidium iodide (PI) (red in Panel b) with an apoptosis detection kit. Panel c shows the overlapping images of (a) and (b). Magnification, 100×. Scale bar, 100 µm. The A10 cells were analysed with TUNEL assays. Magnification, 200×. Confocal microscopic images are using a black colour to mark the damaged DNA in the nucleus (Panel d). (B) Intracellular ROS of tMock or tCRP‐A10 cells were analysed by flow cytometry using 5 μM H_2_DCFDA and excitation at 488 nM (FL1: green). (C) Mitochondrial ROS of the cells were analysed by flow cytometry with 5 μM mitoSOX™ and excitation at 510 nm (FL2: red). (D) Human VSMCs were cultured with recombinant human CRP (25 μg/24 h) and Ref‐1, NOX2, NOX4, p53, MDM2 and GADPH mRNA expression levels were analysed by RT‐PCR. (E) hVSMCs were pre‐transfected with non‐specific siRNA or CRP siRNA (400 pmole/48 h) before 8 Gy IR exposure of the cells. CRP, Ref‐1, STAT3 and phosphorylated STAT3 protein levels were detected with IB. Data are representative of three independent experiments. (F) Quantification was carried out using Quantity One software and unpaired *t*‐tests (**p < 0.01; *p < 0.05)

Our previous study revealed that CRP, as a ligand of the Fcγ receptor IIA (FcγRIIA), can induce VSMCs apoptosis via ROS production by NOX4.^17^ To assess the effect of CRP accumulation, human CRP was overexpressed in A10 cells (tCRP) (Figure S2). At 24‐hours’ post‐transfection, the cells were analysed to detect apoptosis (Figure S3). Confocal microscopy showed that tCRP critically induced apoptosis and nucleic DNA damage in A10 cells (Figure 3A). The tCRP‐A10 cells showed increased production of intracellular ROS (705.4% ± 66.2% compared to 100% ± 15.1% for control tMOCK‐A10 cells) (Figure 3B). The mean value of specific mitochondrial ROS was also shifted from 9.14 (tMock) to 16.4 (tCRP) in A10 cells (Figure 3C). To confirm that CRP protein leads to ROS production, mRNA levels of redox‐related genes were detected in human VSMCs treated with human recombinant CRP (hrCRP). hrCRP enhanced the mRNA levels of NOX2, NOX4, Ref‐1 and p53 compared with the control (Figure 3D). In addition, 8 Gy IR exposure significantly increased the expression levels of CRP and Ref‐1, as well as decreased phosphorylation of STAT3, which was attenuated by CRP knockdown using siRNA CRP (Figure 3E,F). IR can increase CRP expression. *De novo* CRP may trigger apoptosis of VSMC through ROS generation.

### 3.4 Overexpressed CRP protein disrupts STAT3/Ref‐1 complex and alters its expression levels in cytoplasmic and nuclear fractions

**FIGURE 4:**
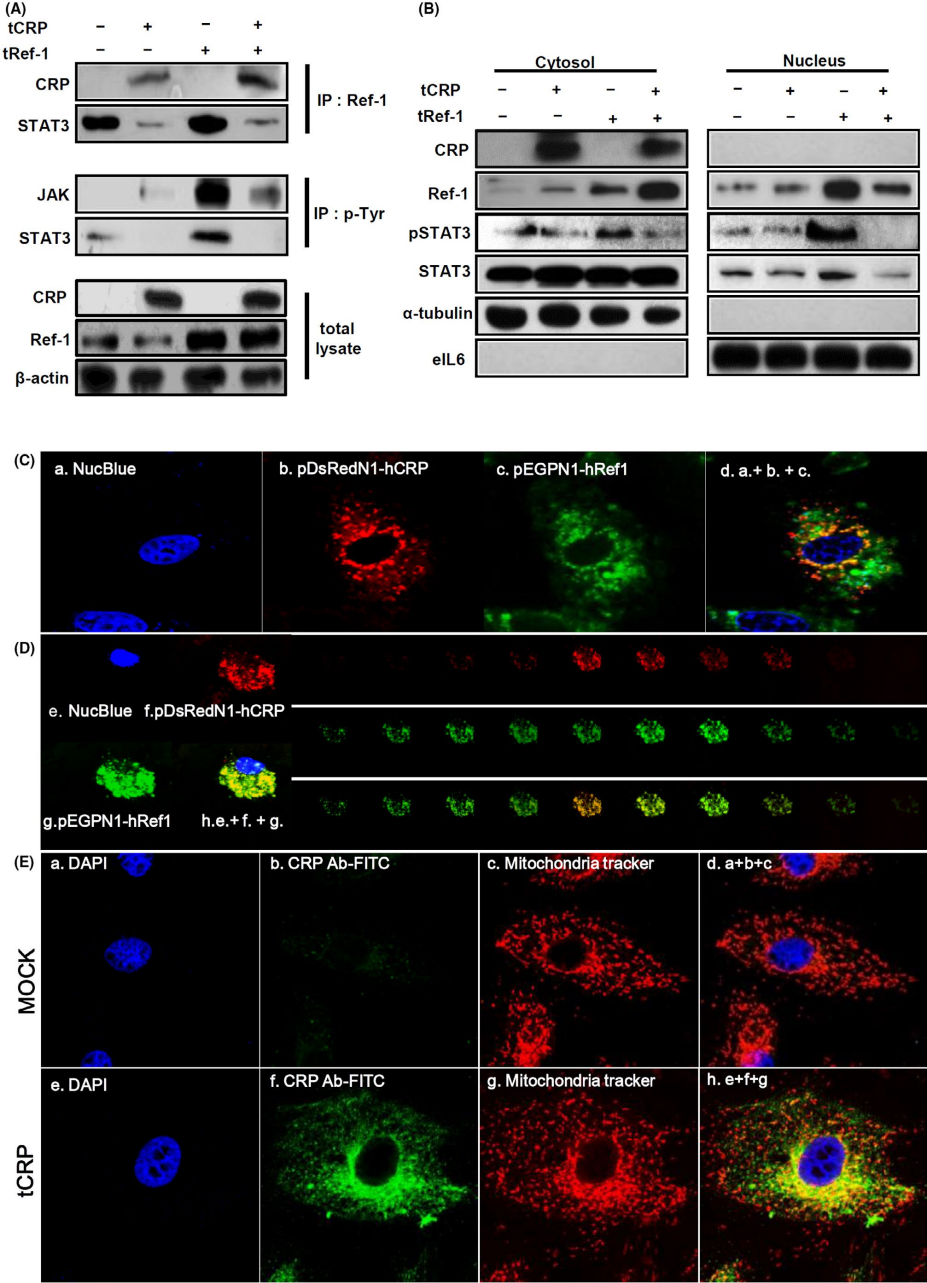
Overexpressed CRP interferes with JAK/STAT3 activity via CRP/Ref‐1 complex formation in VSMCs. A10 were transfected with tMOCK, tCRP, pcDNA3.1‐human Ref‐1 cDNA (tRef‐1), pDsRedN1‐hCRP cDNA or pEGFPN1‐hRef‐1 cDNA for 48 h. (A) Total level of CRP and Ref‐1 protein was detected by IB. Total lysates of the A10 cells were cross‐linked and immunoprecipitated by goat‐anti ref‐1 or rabbit‐anti p‐Tyrosine antibodies. Ref‐1 or phosphorylated Tyrosine antibody‐linked proteins were analysed by IB using mouse anti‐CRP, anti‐JAK and anti‐STAT3 antibodies. (B) Nuclear and cytoplasm extracts were analysed by IB for the specific protein levels, as indicated (CRP, Ref‐1, STAT3‐p, STAT3, tubulin: cytosol marker and eIL6: nucleus marker). The relative band intensities are indicated below each representative IB picture. (C) Localization of CRP and Ref‐1 in A10 cells was analysed by IF. Magnification, 630×. The nucleus was stained with NucBlue™ in living cells (panel a: the nucleus is blue). Expression pattern of Ref‐1 and CRP was shown using confocal microscopic images (panels b and d: CRP is TIRTC red; panels c and d: Ref‐1 is FITC green). (D) Magnification, 630×. Serial images of confocal microscopy were collected at different levels perpendicular to the optical axis (the Z‐stack axis) within a specimen (Panel e: nucleus is blue, panel f: CRP is TRITC red, panel g: Ref‐1 is FITC green, panel h: the merged images of e, g and f). (E) A10 cells were transfected with tMOCK or tCRP for 48 h. Localization of mitochondria and overexpressed CRP protein in A10 cells was analysed through IF using MitoTracker, rabbit‐anti‐CRP antibody and FITC‐conjugated secondary anti‐rabbit antibody. Nuclei were stained with DAPI. Magnification, 630×. CRP expression pattern and localization of mitochondria are shown using confocal microscopic images (panels a and e: the nucleus is blue, panels b and f: CRP is green at 488 nm, panel c and g: mitochondria is red at 578 nm)

Ref‐1 can bind to STAT3 and act as its transcriptional co‐factor in the nucleus.^20^, ^23^, ^24^ To investigate possible changes in the formation of Ref‐1 related protein complexes, IP was performed using a specific Ref‐1 antibody. When overexpressed CRP was bound to Ref‐1, STAT3 detached from Ref‐1 (Figure 4A). To confirm phosphorylation of JAK and STAT3 was involved in their activation, IP was next performed using a phosphorylated tyrosine antibody. Only overexpressed Ref‐1 (tRef‐1) increased the phosphorylated tyrosine levels of JAK and STAT3 compared with tCRP and tRef‐1 co‐transfected VSMCs (Figure 4A). The overexpressed CRP was only found in the cytoplasm (Figure 4B). However, the tRef‐1 alone was overexpressed in the nucleic compartment following STAT3 phosphorylation. In contrast, when CRP and Ref‐1 were co‐overexpressed, the levels of Ref‐1 and phosphorylated STAT3 were markedly reduced in the nucleus (Figure 4B). To determine the subcellular localization of CRP and Ref‐1 in VSMCs, red fluorescent protein (RFP)‐tagged CRP and green fluorescent protein (GFP)‐tagged Ref‐1 were co‐overexpressed and live VSMCs were observed by confocal microscopy. CRP and Ref‐1 were co‐localized in cytoplasm (Figure 4C). These proteins were detected at different levels perpendicular to the optical axis (the *Z*‐axis) from serial sequential sections. They were also sequentially merged on the depth of the extra‐nucleus region in VSMCs (Figure 4D). Overexpressed CRP and Ref‐1 were co‐localized with mitochondria or closely adjacent to mitochondria in VSMCs (Figure 4E, Figure S4). These findings suggest that IR‐induced Ref‐1/CRP complex formation in the cytosol might inhibit STAT3 activity and its translocation to the nucleus promotes STAT3 dissociation from Ref‐1.

### 3.5 Complex of CRP and Ref‐1 leads to mitochondrial dysfunction

**FIGURE 5:**
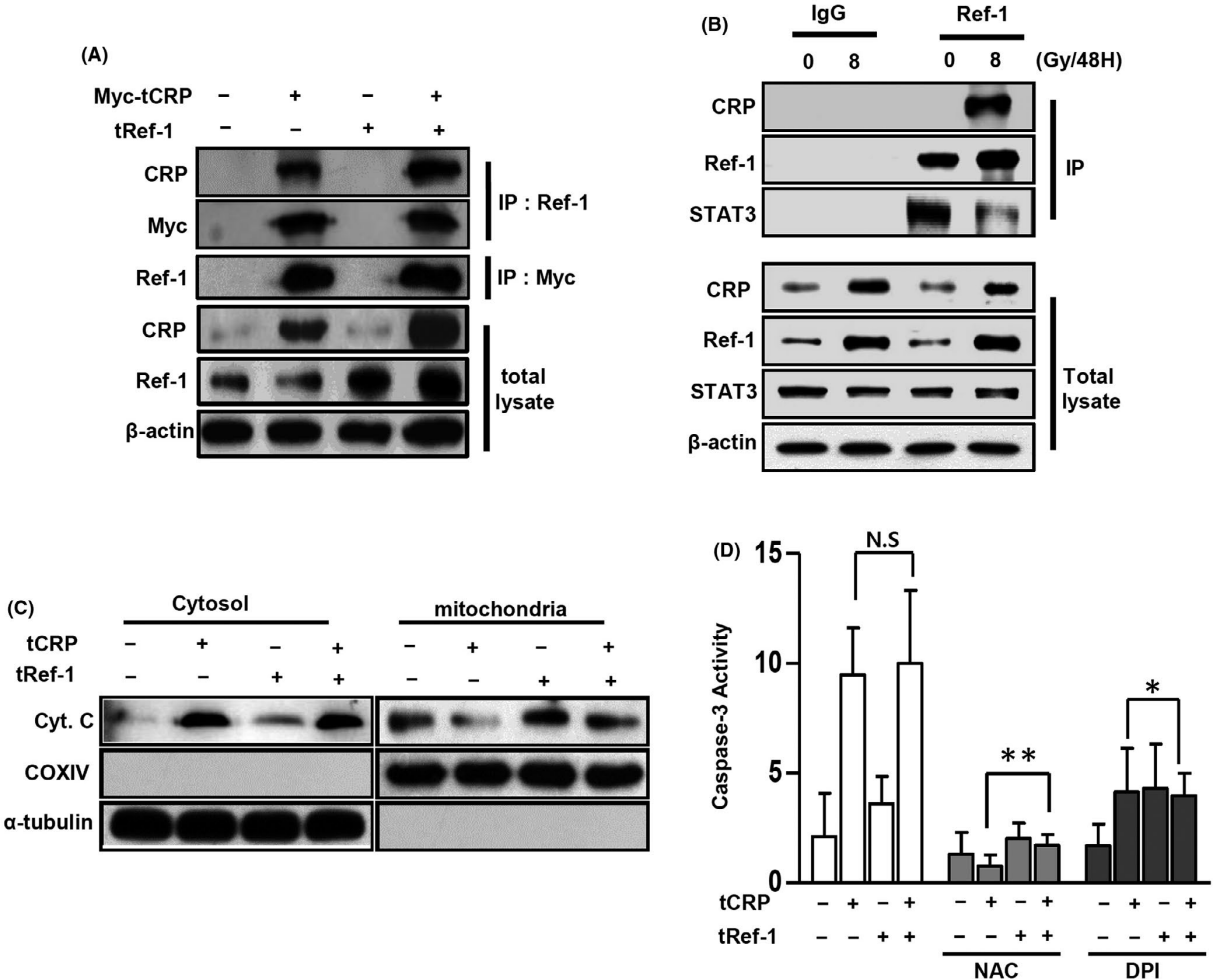
CRP leads to dissociation of the STAT3/Ref‐1 complex and mitochondrial dysfunction in VSMCs. (A, C, D) A10 cells were transfected with pEF1α‐myc vector (myc‐tMOCK), tMOCK, pEF1α‐myc‐hCRP cDNA (myc‐tCRP), tCRP or tRef‐1 for 48 h. Total level of CRP and Ref‐1 protein was detected by IB. Total lysates of the A10 cells were cross‐linked and immunoprecipitated by goat‐anti ref‐1 or rabbit‐anti myc antibodies. Ref‐1 antibody‐linked or myc‐peptide tagged proteins were analysed by IB using mouse anti‐CRP, anti‐Myc and anti‐Ref‐1 antibodies. (B) CRP, Ref‐1 and STAT3 protein levels were detected with IB in total lysates of 8 Gy IR‐exposed hVSMCs. Total cell extracts were immunoprecipitated by IgG or goat‐anti ref‐1 antibodies. IgG or Ref‐1 antibody‐linked proteins were analysed by IB using mouse anti‐CRP, anti‐Ref‐1 and anti‐STAT3 antibodies. (C) Fractions of the cytoplasm and mitochondria were isolated from tCRP‐ or tRef‐1‐A10 cells. Each fraction is shown by the specific antibodies (COXIV: mitochondrial marker, α tubulin: cytosolic marker). Released cytochrome C protein was detected from fractions of cytoplasm and mitochondria using IB. (D) tCRP or tRef‐1 induced caspase‐3 activity was measured by using Vybrant^®^ FAM caspase‐3 assay kits on IR‐exposed A10 cells. The cells were treated with DPI (1 µM) or NAC (0.5 mM) before IR exposure. The mean ± SD value of excitation at 488 nm with unpaired *t*‐tests (**p < 0.01; * *p* < 0.05)

To confirm CRP binding with Ref‐1, lysates of Myc‐tagged CRP‐overexpressed A10 cells were immunoprecipitated with specific anti‐Ref‐1 and anti‐Myc antibodies. As shown in Figure 5A, overexpressed myc‐tagged CRP protein successfully bound to Ref‐1 (Figure S5). IR‐induced CRP protein reduced STAT3 binding levels by binding to Ref‐1, but no difference was observed with a non‐specific IgG antibody (Figure 5B). CRP‐induced apoptosis of VSMCs depends on ROS production by NOX4.^17^ To evaluate whether CRP‐induced mitochondrial dysfunction, tCRP‐ or tRef‐1‐A10 cells were analysed for cytochrome C. As shown in Figure 5C, cytochrome C was critically released to the cytosol in tCRP‐A10 cells. The activation of caspase‐3 by co‐overexpression of CRP and Ref‐1 was increased about five‐fold compared with control or overexpressed Ref‐1 alone (Figure 5D). The increased caspase‐3 activity was attenuated by inhibiting ROS production using 1 μM DPI or 500 μM NAC. DPI significantly blocked caspase‐3 activity by acting simultaneously on CRP and Ref‐1 (Figure 5D). These results suggest that CRP and Ref‐1 complex formation in VSMCs might increase apoptosis via significant mitochondrial dysfunction by ROS.

### 3.6 The CRP/Ref‐1 complex changes cause VSMCs death via cytoplasmic p53

**FIGURE 6:**
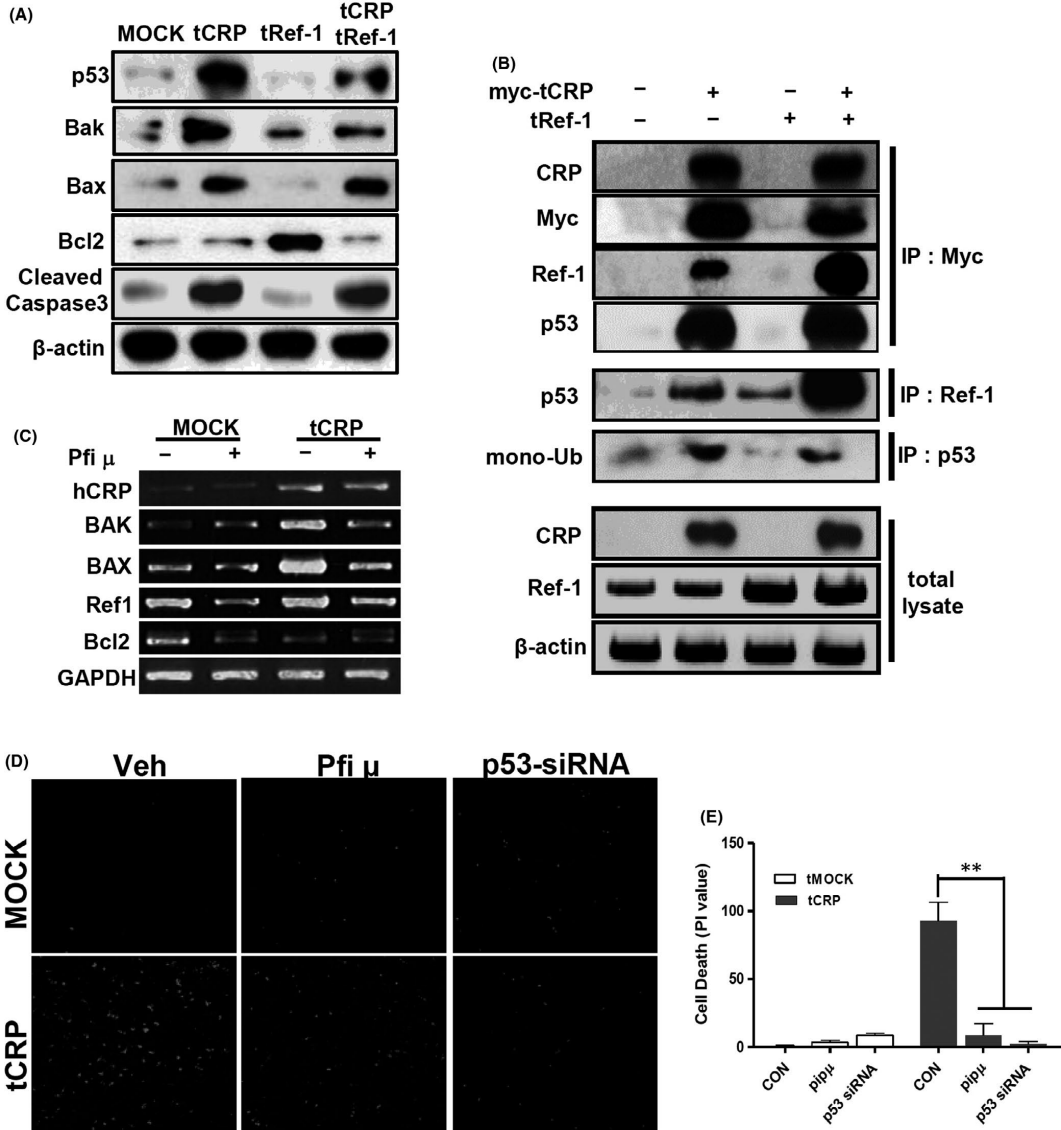
CRP associated‐Ref‐1 induces apoptosis through p53 binding in the cytoplasm of VSMCs. (A, B) A10 cells were transfected with pEF1α‐myc vector (myc‐tMOCK), tMOCK, pEF1α‐myc‐hCRP cDNA (myc‐tCRP), tCRP or tRef‐1 for 48 h. (A) Apoptosis‐related proteins including p53, Bak, Bax, Bcl2 and cleaved caspase‐3 protein levels were detected in total lysates of tMOCK‐, tCRP‐ or tRef‐1‐A10 cells. (B) Total levels of CRP and Ref‐1 protein were detected by IB. Total lysates of the A10 cells were cross‐linked and immunoprecipitated by rabbit‐anti myc, goat‐anti ref‐1 or rabbit‐anti p53 antibodies. CRP, myc, Ref‐1 and p53 protein levels were detected among the myc‐tagged proteins. Ref‐1 antibody‐linked proteins were analysed by IB using p53 antibody. p53 antibody‐linked proteins were analysed by IB using anti‐mono‐ubiquitin antibody. (C, D) tMOCK‐ or tCRP‐A10 cells were treated with PFT µ (5 µM) or transfected with p53 siRNA for 48 h. (C) mRNA levels of CRP and apoptotic genes were analysed by RT‐PCR. (D) Magnification, 100×. Confocal images of cell death (infiltrated PI dye and stained damaged nuclei: red colour). (E) The mean ± SD value of excitation at 585 nm with unpaired *t*‐tests (** *p* < 0.01)

As shown in Figure 6A–C, tCRP in VSMCs increased the expression levels of apoptotic genes, including p53, bax, bak and cleaved caspase‐3, whereas tRef‐1 alone increased Bcl2. This suggests that CRP might change the phenotype of VSMCs. Ref‐1 protein is associated with p53.^18^, ^40^ To examine this possibility, IP was performed using myc‐tagged CRP‐overexpressed VSMCs. The myc‐tagged tCRP induced‐p53 formed a complex with Ref‐1/CRP proteins in VSMCs (Figure 6B). As shown in Figure 4B,C, co‐overexpressed CRP and Ref‐1 in VSMCs were markedly increased in the cytoplasm. Mono‐ubiquitination of p53 causes it to transmigrate from the nucleus to the cytoplasmic compartment.^41^ As shown in Figure 6B, mono‐ubiquitinated p53 was mainly detected in the lane with tCRP‐VSMC. The tRef‐1 alone could not induce mono‐ubiquitination of p53. tCRP induced p53 expression and co‐localized with p53 in the mitochondria or cytoplasm (Figure S6). When p53 migration was blocked using pifithrin μ (PFT μ), an inhibitor of cytoplasmic accumulation of p53,^42^ the expression levels of Bax and Bak were attenuated compared with those in tCRP‐VSMCs (Figure 6C). In addition, confocal images showed that CRP‐induced cell death was blocked by inhibiting cytoplasmic p53 using PFT μ or p53 siRNA (Figure 6D,E). These results suggest that the complex formation of CRP and Ref‐1 in VSMCs might increase apoptosis via the accumulation and association with cytoplasmic p53.

### 3.7 CRP, Ref‐1 and p53 proteins are co‐localized in VSMCs of human coronary plaques

**FIGURE 7:**
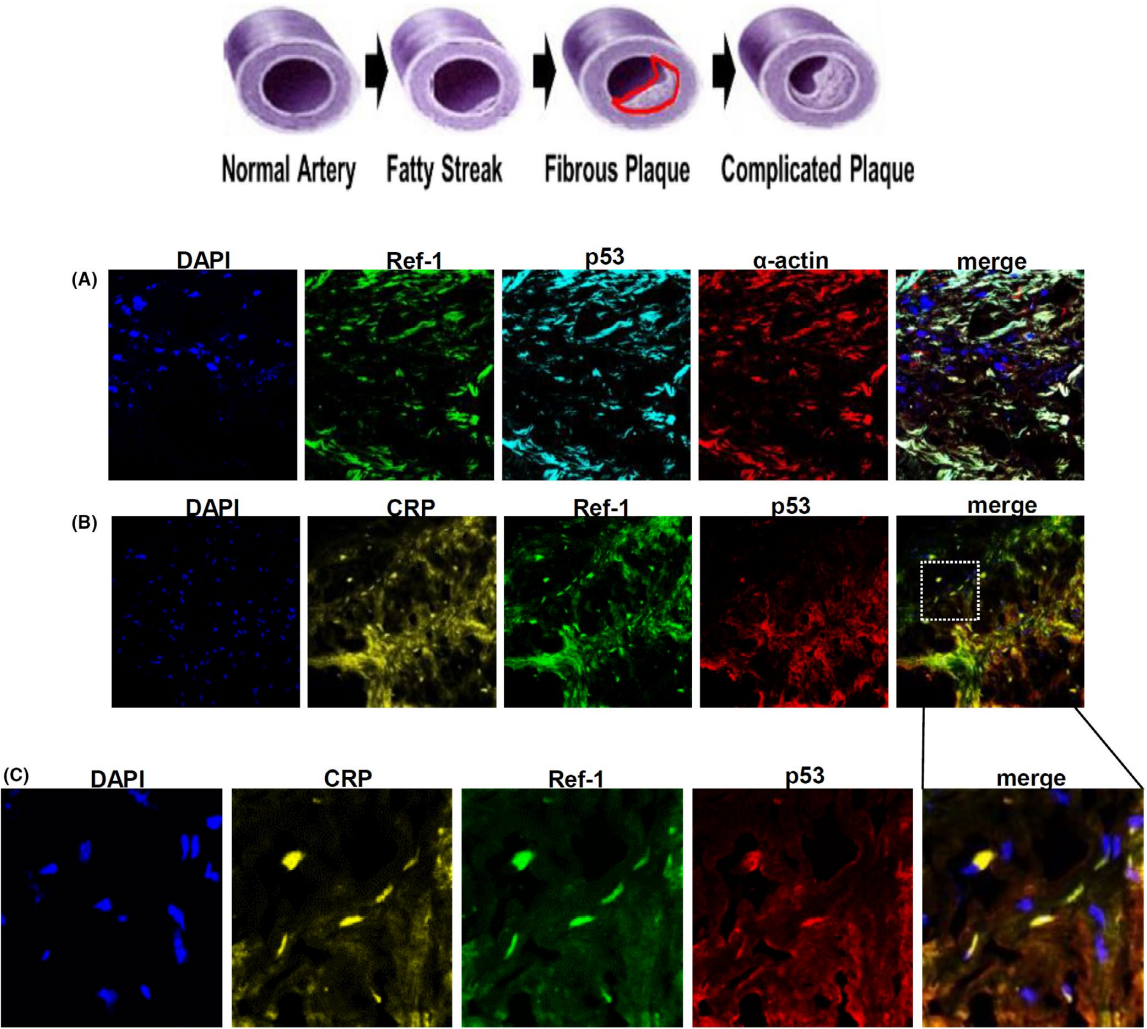
Ref‐1, CRP and p53 proteins co‐localized in VSMCs of human coronary plaque. Human atheromatous plaques were obtained by directional coronary atherectomy (DCA) and were cyto‐static sectioned. (A–C) Tissues were analysed with triple IF using anti‐αSMA (α smooth muscle actin)‐TRITC, p53‐TRITC, Ref‐1‐FITC and CRP‐cy5 antibodies. Nuclei were stained with DAPI. (A) Magnification, 400×. (B) Magnification, 100×. (C) Magnification, 630×

Human atherosclerotic plaques contain abundant VSMCs and macrophages.^43^ The image of immunofluorescence clearly showed that Ref‐1 and p53 were expressed in α‐smooth muscle actin (α‐SMA) (+) VSMCs in human atherosclerotic lesions (Figure 7A). The same area of α‐SMA (+) staining also generally co‐localized with CRP (+), Ref‐1 (+) and p53 (+) areas (Figure 7B,C).

## 4 DISCUSSION

Radiation‐induced CRP initiates and maintains chronic inflammation that causes late radiation complications. The serum CRP level is usually measured from undetectable to 1.0 mg/L in healthy humans. CRP can be de novo synthesized in VSMCs or macrophages within atherosclerotic lesions in inflammatory responses.^44^ Blaschke et al.^15^ reported that CRP can induce apoptosis of human coronary VSMCs by mediating caspase activity. In addition, we have previously reported that CRP can trigger NOX4 activation and consequently induce apoptosis of human aortic VSMCs.^17^ Radiotherapy‐induced CRP can increase the risk of skin toxicity and cardiac morbidity, and ischaemic heart diseases.^45^ In this context, the present study confirmed that IR‐exposed VSMCs produced ROS dose‐dependently even 48 h later. In artificial conditions, overexpressed CRP in VSMCs dramatically increased their intracellular ROS production.

To examine how VSMCs survival could be modulated by de novo synthesis of CRP, hrCRP was used to treat VSMCs. This treatment enhanced the expression levels of the NOX2, NOX4, Ref‐1 and p53 genes. NOX1, NOX2 and NOX4 are the three main NOXs known to produce ROS, which plays key roles as secondary messengers, in human vasculature and endothelial cells.^37^, ^46^ When NOXs produce excessive ROS, they can result in cardiovascular symptoms such as atherosclerosis and instability of atheromatous plaques through cell phenotype modulation.^47^ This background of increased apoptotic VSMCs through IR‐induced CRP can be explained by our results showing that scavenging ROS by NAC in IR‐exposed VSMCs reduced the expression of apoptosis genes. CRP in the region of directional coronary atherosclerosis (DCA) can induce ROS production from p22^phox^‐based NOX.^17^, ^48^ Furthermore, NOX1, NOX2 and NOX4 proteins strictly require the p22^phox^ subunit for their enzyme activity.^28^, ^36^ We generated p22^phox^ knockdown of VSMCs (p22^phox^‐KD) by lentivirus to inhibit NOX activity, which was able to inhibit the induction of p22^phox^ expression by CRP (Figure S7). We also suppressed NOX activity using DPI. Both approaches consistently demonstrated profound inhibition of radiation‐induced apoptosis by attenuating caspase‐3 activity and DNA damage. These results confirmed that radiation‐induced CRP could increase ROS production by NOX, thus leading to apoptosis of VSMCs.

In the early phase of atherosclerosis, VSMCs affects some aspects of plaque progression by unclear mechanisms.^6^, ^45^ Ref‐1 is highly expressed in atherosclerotic lesion and regulated the redox system in an in vivo model of hyper‐homocysteinemia‐accelerated atherosclerosis.^49^ Previous reports suggested that an increased Ref‐1 level may induce VSMC proliferation as a co‐transcriptional factor in the STAT3/Ref‐1 complex.^24^ Sustained expression of Ref‐1 and CRP accumulation in the cytosol may inhibit STAT3/Ref‐1 interactions and increase the inflammatory response of CRP, thus accelerating atherosclerosis after radiotherapy. Apoptosis of VSMCs is a cause of plaque instability and inflammation in the vascular system,^6^ explaining why VSMCs might crucially proceed to apoptosis.

Ref‐1 as a co‐factor can directly bind to and modulate transcription factors including AP‐1, HIF1α, NF‐κB and p53.^50^ Ref‐1 regulates cell growth, apoptosis, the intracellular redox state and mitochondrial function.^51^ In the current study, Ref‐1 expression was increased following IR‐induced ROS production. We also found that de novo‐synthesized CRP or IR‐induced CRP was associated with Ref‐1. Previously, Ref‐1 was shown to regulate the transcription of STAT3 through STAT3 interactions with Ref‐1.^23^ We found that Ref‐1 could bind to STAT3 and induce the activation of JAK/STAT3. However, co‐overexpression of Ref‐1 and CRP changed the Ref‐1 effect from cell survival to apoptosis. The CRP/Ref‐1 complex might provide an important clue about the inflammatory response and VSMCs during the development of atherosclerosis, suggesting a new viewpoint on multifunctional Ref‐1 processing in cardiovascular disorders. JAK/STAT3 signalling is a potential axis of signal transductions that can regulate the proliferation of VSMCs.^52^ Based on the dissociation of STAT3 from Ref‐1 that we observed under 8 Gy exposure of VSMCs, IR‐induced CRP and Ref‐1 complex formation could be linked to the transcriptional repression of STAT3. The activity of STAT3 opposes CRP‐induced apoptosis, at least within VSMCs. The overexpression of Ref‐1 alone upregulated JAK and STAT3 activities, whereas co‐expression of Ref‐1 and CRP dramatically down‐regulated the phosphorylation of JAK and STAT3. When Ref‐1 was co‐overexpressed with CRP, patterns of Ref‐1 and STAT3 localization in intracellular regions were changed. STAT3 was dissociated from Ref‐1 and phosphorylated STAT3 proteins were decreased in the nuclei after overexpressing CRP. Furthermore, in the Ref‐1 capacity of managing ROS production and promoting cell survival, newly accumulated CRP might trigger the switch of the activity of Ref‐1 from proliferation to apoptosis. Formation of the STAT3/Ref‐1 complex and increased phosphorylation of JAK and STAT3 in the nucleus may indicate their involvement in gene targeting and transcription, driving the proliferation of VSMCs. The increase in cytoplasmic CRP under an inflammatory state could also induce a switch to apoptotic cell death via formation of the CRP/Ref‐1 complex. With the co‐overexpression of CRP and Ref‐1 proteins, we indeed observed apoptosis. While the overexpressed Ref‐1 showed signs of cell proliferation such as increased DNA synthesis in S phase (Figure S8) and anti‐apoptotic Bcl2 expression, the co‐overexpressed CRP and Ref‐1 may lead to apoptosis of VSMCs, notably by increasing Bax expression, caspase‐3 activation and releasing cytochrome c. As a result, cell death was found to be induced by newly synthesized CRP alone. In addition to the unilateral effect of CRP, CRP‐induced apoptosis seems to be mediated by regulating endogenous Ref‐1 activity.

p53 is as a transcription factor and increased in cells after exposure to radiation.^53^ A mouse model of overexpressed p53 in advanced atherosclerotic plaques also demonstrated that p53 can lead to the death of smooth muscle cells and induce atherosclerotic plaque destabilization.^54^, ^55^ In the current study, in vitro tCRP or hrCRP critically increased p53 expression in VSMCs. Ref‐1 is associated with p53 and it regulates pro‐apoptotic p53 both in vivo and in vitro.^40^ In this study, when CRP and Ref‐1 were co‐overexpressed in VSMCs showed that increased p53 was associated with the CRP‐Ref‐1 complex. Mitochondrial damage and dysfunction can accelerate atherosclerosis and rupture of plaques by enhancing VSMCs apoptosis.^56^ Here, we showed that overexpressed CRP of VSMCs dramatically enhanced p53 expression and the proportion of mono‐ubiquitinated p53 under the same conditions. Mono‐ubiquitination of p53 triggers its export from the nucleus.^41^ Nuclear exported p53 can accumulate in the cytoplasm before transcription‐independent p53 apoptosis via mitochondrial dysfunction, cytochrome c release and procaspase‐3 activation.^41^, ^42^ When mono‐ubiquitinated p53 was associated with the CRP/Ref‐1 complex in VSMCs, we found that the expression levels of apoptotic genes, including Bax, Bak, cleaved caspase‐3 and cytosolic cytochrome C were increased. Exporting p53 from the nucleus might play a pro‐apoptotic role. However, pretreatment with PFT μ or p53 siRNA, an inhibitor of mitochondrial p53 accumulation and function,^42^ inhibited apoptotic genes expression by tCRP. Thus, we observed that during the switch from VSMC survival to cell death, formation of the CRP‐Ref‐1 complex in the cytoplasm in the presence of cytoplasmic p53 resulted in apoptosis via a transcription‐independent pathway. During atherosclerotic plaque development, involving Ref‐1 and ROS production via accumulation of CRP, p53 might induce apoptosis in VSMC cells, thus causing unstable plaques.

CRP is synthesized by VSMCs or macrophages within severe atherosclerotic plaque lesions.^17^ CRP is frequently detected in human unstable or ruptured plaques. It is particularly prominent in VSMCs as a major factor involved in thinning of the fibrotic cap.^44^, ^48^ In DCA, the same tissue used for staining in reference number 17 for our studies, the overlapping area of CRP and Ref‐1 staining was spread on α‐actin‐positive VSMCs. CRP and Ref‐1 proteins were found to be co‐localized and generally existed in the cytoplasm rather than in the nucleus. The observation of co‐localization of CRP and Ref‐1 in human DCA thus supports our hypothesis. It is unclear what role CRP plays in the transition between VSMC proliferation and death during atherosclerosis. Our study showed that changes in Ref‐1/STAT3 activity induced by CRP accumulation were closely related to the induction of atherosclerotic plaque instability. We can propose that the CRP/Ref‐1 complex is one of the causative factors in switching the VSMCs phenotype, which determines the development of unstable plaques. In other words, our findings suggest that the expression and accumulation of CRP could tune Ref‐1 activity, leading to the death of VSMC with consequences on unstable plaque formation. The molecular mechanisms involved in the early‐to‐late chronic inflammatory stages of atherosclerosis remain elusive. Furthermore, the signalling mechanisms of vascular damage caused by radiotherapy are even more ambiguous. For therapeutic applications, molecular mechanisms related to other phenotypes such as migration, invasion and silencing of blood or vascular cells should be further elucidated. Based on the overall results of the current study, it is expected that studying the transformation of the VSMC phenotype that induces vascular damage through CRP inhibition and modulation of Ref‐1 activity will help to improve radiation therapy and prevent its side effects.

## CONFLICT OF INTEREST

The authors confirm that there is no conflict of interest.

## AUTHOR CONTRIBUTIONS

**Je‐Won Ryu:** Conceptualization (equal); data curation (equal); formal analysis (equal); investigation (equal); methodology (equal); project administration (equal); validation (equal); writing – original draft (equal); writing – review and editing (equal). **In‐Hye Jung:** Conceptualization (equal); data curation (equal); formal analysis (equal); investigation (equal); methodology (equal); project administration (equal); software (equal); validation (equal); writing – original draft (equal). **Eun‐Young Park:** Conceptualization (equal); data curation (equal); formal analysis (equal); investigation (equal); methodology (equal); project administration (equal); software (equal); validation (equal); writing – original draft (equal). **Kang‐Hyun Kim:** Data curation (equal); methodology (equal); validation (equal); visualization (equal). **Kyunggon Kim:** Funding acquisition (equal); investigation (equal); project administration (equal); resources (equal). **Jeonghun Yeom:** Formal analysis (equal); investigation (equal); software (equal). **Jinhong Jung:** Conceptualization (equal); data curation (equal); formal analysis (equal); funding acquisition (equal); project administration (equal); supervision (equal); writing – review and editing (equal). **Sang‐wook Lee:** Conceptualization (equal); data curation (equal); formal analysis (equal); funding acquisition (equal); methodology (equal); project administration (equal); supervision (equal); writing – review and editing (equal).

## Supporting information

Supplementary Material

Supplementary Material

Supplementary Material

Supplementary Material

Supplementary Material

Supplementary Material

Raw data Fig S7a

Raw data Fig S7a

## Data Availability

All data generated or analysed during this study are included in this published article and its supplementary information files.

## References

[1] Venkatesulu BP , Mahadevan LS , Aliru ML , et al. Radiation‐induced endothelial vascular injury: a review of possible mechanisms. JACC Basic Transl Sci. 2018;3(4):563‐572. doi:10.1016/j.jacbts.2018.01.014 30175280 PMC6115704

[2] Kim WL , Seo S , Kim D , et al.Cellular stress responses in radiotherapy. Cells. 2019;8(9):1105. 10.3390/cells8091105 PMC6769573 31540530

[3] Groarke JD , Nguyen PL , Nohria A , Ferrari R , Cheng S , Moslehi J . Cardiovascular complications of radiation therapy for thoracic malignancies: the role for non‐invasive imaging for detection of cardiovascular disease. Eur Heart J. 2014;35(10):612‐623. doi:10.1093/eurheartj/eht114 23666251 PMC3945797

[4] Libby P , Ridker PM , Hansson GK . Progress and challenges in translating the biology of atherosclerosis. Nature. 2011;473(7347):317‐325. doi:10.1038/nature10146 21593864

[5] Virmani R , Kolodgie FD , Burke AP , Farb A , Schwartz SM . Lessons from sudden coronary death. Arterioscler Thromb Vasc Biol. 2000;20(5):1262‐1275. 10.1161/01.atv.20.5.1262 10807742

[6] Bennett MR , Sinha S , Owens GK . Vascular smooth muscle cells in atherosclerosis. Circ Res. 2016;118(4):692‐702. doi:10.1161/CIRCRESAHA.115.306361 26892967 PMC4762053

[7] Clarke M , Bennett M . The emerging role of vascular smooth muscle cell apoptosis in atherosclerosis and plaque stability. Am J Nephrol. 2006;26(6):531‐535. doi:10.1159/000097815 17159340

[8] Clarke MC , Figg N , Maguire JJ , et al. Apoptosis of vascular smooth muscle cells induces features of plaque vulnerability in atherosclerosis. Nat Med. 2006;12(9):1075‐1080. doi:10.1038/nm1459 16892061

[9] Blakely WF . Early biodosimetry response: recommemdations for mass‐casualty radiation accidents and teerorism [Report of biodosimetry project]. Armed Forces Radiology Research Institute (AFRRI). 2008;1(1):1‐27. https://www.researchgate.net/publication/228624567. Accessed January 1, 2008.

[10] Ossetrova NI , Sandgren DJ , Blakely WF . C‐reactive protein and serum amyloid A as early‐phase and prognostic indicators of acute radiation exposure in nonhuman primate total‐body irradation model. Radiat Meas. 2011;46:1019‐1024. doi:10.1016/j.radmeas.2011.05.021

[11] Carsten Nieder BM , Dalhaug A , Pawinski A , Haukland E . Palliative radiotherapy in cancer patients with increased serum C‐reactive protein level. In vivo. 2016;30(5):581‐586. 27566075

[12] Silja Norja LN , Karhunen PJ , Goebeler S C‐reactive protein in vulnerable coronary plaques. J Clin Pathol. 2006;60(5):545‐548. doi:10.1136/jcp.2006.038729 16790690 PMC1994542

[13] Allen P , Burke RPT , Kolodgie F , et al. Elevated C‐reactive protein values and atherosclerosis in sudden coronary death association with different pathologies. Circulation. 2002;105:2019‐2023. doi:10.1161/01.CIR.0000015507.29953.38 11980679

[14] Hong MK , Mintz GS , Lee CW , et al. Comparison of coronary plaque rupture between stable angina and acute myocardial infarction: a three‐vessel intravascular ultrasound study in 235 patients. Circulation. 2004;110(8):928‐933. doi:10.1161/01.CIR.0000139858.69915.2E 15313951

[15] Blaschke F , Bruemmer D , Yin F , et al. C‐reactive protein induces apoptosis in human coronary vascular smooth muscle cells. Circulation. 2004;110(5):579‐587. doi:10.1161/01.CIR.0000136999.77584.A2 15277326

[16] Xu S , Chamseddine AH , Carrell S , Miller FJ Jr . Nox4 NADPH oxidase contributes to smooth muscle cell phenotypes associated with unstable atherosclerotic plaques. Redox Biol. 2014;2:642‐650. doi:10.1016/j.redox.2014.04.004 24936437 PMC4052526

[17] Ryu J , Lee CW , Shin JA , et al. FcgammaRIIa mediates C‐reactive protein‐induced inflammatory responses of human vascular smooth muscle cells by activating NADPH oxidase 4. Cardiovasc Res. 2007;75(3):555‐565. doi:10.1016/j.cardiores.2007.04.027 17531211

[18] Tell G , Damante G , Caldwell D , Kelley MR . The intracellular localization of APE1/Ref‐1: more than a passive phenomenon? Antioxid Redox Signal. 2005;7(3–4):367‐384. doi:10.1089/ars.2005.7.367 15706084

[19] Choi S , Joo HK , Jeon BH . Dynamic regulation of APE1/Ref‐1 as a therapeutic target protein. Chonnam Med J. 2016;52(2):75‐80. doi:10.4068/cmj.2016.52.2.75 27231670 PMC4880582

[20] Thakur S , Sarkar B , Cholia RP , Gautam N , Dhiman M , Mantha AK . APE1/Ref‐1 as an emerging therapeutic target for various human diseases: phytochemical modulation of its functions. Exp Mol Med. 2014;46(7):e106. doi:10.1038/emm.2014.42 25033834 PMC4119211

[21] Lee YR , Joo HK , Lee E‐O , et al. Plasma APE1/Ref‐1 correlates with atherosclerotic inflammation in ApoE−/− mice. Biomedicines. 2020;8(9):1‐16. doi:10.3390/biomedicines8090366 PMC7555038 32967121

[22] He T , Weintraub NL , Goswami PC , et al. Redox factor‐1 contributes to the regulation of progression from G0/G1 to S by PDGF in vascular smooth muscle cells. Am J Physiol Heart Circ Physiol. 2003;285(2):H804‐H812. doi:10.1152/ajpheart.01080.2002 12730053

[23] Ray S , Lee C , Hou T , Bhakat KK , Brasier AR . Regulation of signal transducer and activator of transcription 3 enhanceosome formation by apurinic/apyrimidinic endonuclease 1 in hepatic acute phase response. Mol Endocrinol. 2010;24(2):391‐401. doi:10.1210/me.2009-0319 20032196 PMC2817606

[24] Cardoso AA , Jiang Y , Luo M , et al. APE1/Ref‐1 regulates STAT3 transcriptional activity and APE1/Ref‐1‐STAT3 dual‐targeting effectively inhibits pancreatic cancer cell survival. PLoS One. 2012;7(10):e47462. doi:10.1371/journal.pone.0047462 23094050 PMC3477158

[25] Angkeow P , Deshpande S , Qi B , et al. Redox factor‐1: an extra‐nuclear role in the regulation of endothelial oxidative stress and apoptosis. Cell Death Differ. 2002;9(7):717‐725. doi:10.1038/sj.cdd.4401025 12058277

[26] Zhang T , Zhang X , Yu W , et al. Effects of chemokine‐like factor 1 on vascular smooth muscle cell migration and proliferation in vascular inflammation. Atherosclerosis. 2013;226(1):49‐57. doi:10.1016/j.atherosclerosis.2012.09.023 23102782

[27] Cai Y , Knight WE , Guo S , Li JD , Knight PA , Yan C . Vinpocetine suppresses pathological vascular remodeling by inhibiting vascular smooth muscle cell proliferation and migration. J Pharmacol Exp Ther. 2012;343(2):479‐488. doi:10.1124/jpet.112.195446 22915768 PMC3477207

[28] Adane B , Ye H , Khan N , et al. The hematopoietic oxidase NOX2 regulates self‐renewal of leukemic stem cells. Cell Rep. 2019;27(1):238‐254.e6. doi:10.1016/j.celrep.2019.03.009 30943405 PMC6931909

[29] Ryu JW , Hong KH , Maeng JH , et al. Overexpression of uncoupling protein 2 in THP1 monocytes inhibits beta2 integrin‐mediated firm adhesion and transendothelial migration. Arterioscler Thromb Vasc Biol. 2004;24(5):864‐870. doi:10.1161/01.ATV.0000125705.28058.eb 15016641

[30] Park S , Park J‐H , Ryu S‐H , et al. Radiation‐induced phosphorylation of serine 360 of SMC1 in human peripheral blood mononuclear cells. Radiat Res. 2019;191(3):262‐270. doi:10.1667/RR15179.1 30702968

[31] Tell G , Scaloni A , Pellizzari L , Formisano S , Pucillo C , Damante G . Redox potential controls the structure and DNA binding activity of the paired domain. J Biol Chem. 1998;273(39):25062‐25072. doi:10.1074/jbc.273.39.25062 9737963

[32] Kawamura K , Qi F , Kobayashi J . Potential relationship between the biological effects of low‐dose irradiation and mitochondrial ROS production. J Radiat Res. 2018;59(Suppl 2):ii91‐ii97. doi:10.1093/jrr/rrx091 29415254 PMC5941154

[33] Yuri Sakai TY , Yoshikawa Y , Bo T , et al. NADPH oxidase 4 mediates ROS production in radiation‐induced senescent cells and promotes migration of inflammatory cells. Free Radical Res. 2018;52(1):92‐102. doi:10.1080/10715762.2017.1416112 29228832

[34] Blakely WF , Ossetrova NI , Whitnall MH , et al. Multiple parameter radiation injury assessment using a nonhuman primate radiation model‐biodosimetry applications. Health Phys. 2010;98(2):153‐159. doi:10.1097/HP.0b013e3181b0306d 20065677

[35] Luan YY , Yao YM . The clinical significance and potential role of C‐reactive protein in chronic inflammatory and neurodegenerative diseases. Front Immunol. 2018;9:1302. doi:10.3389/fimmu.2018.01302 29951057 PMC6008573

[36] Prior KK , Leisegang MS , Josipovic I , et al. CRISPR/Cas9‐mediated knockout of p22phox leads to loss of Nox1 and Nox4, but not Nox5 activity. Redox Biol. 2016;9:287‐295. doi:10.1016/j.redox.2016.08.013 27614387 PMC5021817

[37] Clempus RE , Griendling KK . Reactive oxygen species signaling in vascular smooth muscle cells. Cardiovasc Res. 2006;71(2):216‐225. doi:10.1016/j.cardiores.2006.02.033 16616906 PMC1934427

[38] Yokoyama M , Inoue N , Kawashima S . Role of the vascular NADH/NADPH oxidase system in atherosclerosis. Ann N Y Acad Sci. 2000;902(1):241‐248. doi:10.1111/j.1749-6632.2000.tb06319.x 10865844

[39] Ambasta RK , Kumar P , Griendling KK , Schmidt HH , Busse R , Brandes RP . Direct interaction of the novel Nox proteins with p22phox is required for the formation of a functionally active NADPH oxidase. J Biol Chem. 2004;279(44):45935‐45941. doi:10.1074/jbc.M406486200 15322091

[40] Gaiddon C , Moorthy NC , Prives C . Ref‐1 regulates the transactivation and pro‐apoptotic functions of p53 in vivo. EMBO J. 1999;18(20):5609‐5621. doi:10.1093/EMBOJ/18.20.5609 10523305 PMC1171629

[41] Brooks CLL , Li Muyang , Gu Wei . Monoubiquitination: the signal for p53 nuclear export? Cell Cycle. 2004;3(4):434‐436. doi:10.4161/cc.3.4.782 14976431

[42] Chiu GS , Maj MA , Rizvi S , et al. Pifithrin‐mu prevents cisplatin‐induced chemobrain by preserving neuronal mitochondrial function. Cancer Res. 2017;77(3):742‐752. doi:10.1158/0008-5472.CAN-16-1817 27879267 PMC5290207

[43] Tabas I , Garcia‐Cardena G , Owens GK . Recent insights into the cellular biology of atherosclerosis. J Cell Biol. 2015;209(1):13‐22. doi:10.1083/jcb.201412052 25869663 PMC4395483

[44] Paolo Calabró JTW , Yeh ETH . Inflammatory cytokines stimulated C‐reactive protein production by human coronary artery smooth muscle cells. Circulation. 2003;108:1930‐1932. doi:10.1161/01.CIR.0000096055.62724.C5 14530191

[45] Hu JJ , Urbanic JJ , Case LD , et al. Association between inflammatory biomarker C‐reactive protein and radiotherapy‐induced early adverse skin reactions in a multiracial/ethnic breast cancer population. J Clin Oncol. 2018;36(24):2473‐2482. doi:10.1200/JCO.2017.77.1790 29989859 PMC6097833

[46] Burtenshaw D , Hakimjavadi R , Redmond EM , Nox CPA . Nox, reactive oxygen species and regulation of vascular cell fate. Antioxidants. 2017;6(4):90‐ 10.3390/antiox6040090 PMC5745500 29135921

[47] Cave A . Selective targeting of NADPH oxidase for cardiovascular protection. Curr Opin Pharmacol. 2009;9(2):208‐213. doi:10.1016/j.coph.2008.10.001 18973829

[48] Kobayashi S , Inoue N , Ohashi Y , et al. Interaction of oxidative stress and inflammatory response in coronary plaque instability: important role of C‐reactive protein. Arterioscler Thromb Vasc Biol. 2003;23(8):1398‐1404. doi:10.1161/01.ATV.0000081637.36475.BC 12805076

[49] Dai J , Li W , Chang L , et al. Role of redox factor‐1 in hyperhomocysteinemia‐accelerated atherosclerosis. Free Radic Biol Med. 2006;41(10):1566‐1577. doi:10.1016/j.freeradbiomed.2006.08.020 17045925

[50] Bhakat KK , Mantha AK , Mitra S . Transcriptional regulatory functions of mammalian AP‐endonuclease (APE1/Ref‐1), an essential multifunctional protein. Antioxid Redox Signal. 2009;11(3):621‐638. doi:10.1089/ARS.2008.2198 18715144 PMC2933571

[51] Vascotto C , Cesaratto L , Zeef LA , et al. Genome‐wide analysis and proteomic studies reveal APE1/Ref‐1 multifunctional role in mammalian cells. Proteomics. 2009;9(4):1058‐1074. doi:10.1002/pmic.200800638 19180539 PMC3802553

[52] Wang YC , Cui XB , Chuang YH , Chen SY . Janus kinase 3, a novel regulator for smooth muscle proliferation and vascular remodeling. Arterioscler Thromb Vasc Biol. 2017;37(7):1352‐1360. doi:10.1161/ATVBAHA.116.308895 28473442 PMC5503700

[53] Fei P , El‐Deiry WS . P53 and radiation responses. Oncogene. 2003;22(37):5774‐5783. doi:10.1038/sj.onc.1206677 12947385

[54] von der Thusen JH , van Vlijmen BJ , Hoeben RC , et al. Induction of atherosclerotic plaque rupture in apolipoprotein E‐/‐ mice after adenovirus‐mediated transfer of p53. Circulation. 2002;105(17):2064‐2070. doi:10.1161/01.cir.0000015502.97828.93 11980686

[55] Zhang L , Liu Y , Lu XT , et al. Intraplaque injection of Ad5‐CMV.p53 aggravates local inflammation and leads to plaque instability in rabbits. J Cell Mol Med. 2009;13(8B):2713‐2723. doi:10.1111/j.1582-4934.2008.00538.x 19602038 PMC6512386

[56] Madamanchi NR , Runge MS . Mitochondrial dysfunction in atherosclerosis. Circ Res. 2007;100(4):460‐473. doi:10.1161/01.RES.0000258450.44413.96 17332437

