# Supplementary material for: Radiation‐induced C‐reactive protein triggers apoptosis of vascular smooth muscle cells through ROS interfering with the STAT3/Ref‐1 complex
Source: J Cell Mol Med. 2022 Feb 17;26(7):2104–18. doi: 10.1111/jcmm.17233 (PMC8980952; doi:10.1111/jcmm.17233)
Supplement: Supplementary file 1 — Supplementary Material [file JCMM-26-2104-s008.docx]

**Supplement Information**

**Radiation-induced C-reactive protein triggers apoptosis of vascular smooth muscle cells through ROS interfering with the STAT3/Ref-1 complex**

Jewon Ryu^a^**^†^**, In Hye Jung^b^**^†^**, Eun-Young Park^c^**^†^**, Kang Hyun Kim^a^, Kyunggon Kim^a^, Junghun Yeoum^a^, Jinhong Jung^c^**^‡^**, and Sang-wook Lee^c^**^‡^**

**SUPPLEMENTARY MATERIALS AND METHODS**

**Generation of oligodeoxynucleotides (ODN) of p22^phox^**

Phosphothioate-modified ODN specific for p22^phox^ binding sites was used to inhibit the activities of p22^phox^ containing NADPH oxidase as previously described [[26](#_ENREF_26)]. The nucleotide sequences of dumbbell-type were phosphothioated and double-stranded ODN against the p22^phox^ binding sites and their mismatched ODNs as follows: p22^phox^ -specific decoy ODN, 5'-GAT CTG CCC CAT GGT GAG GAC C-3'; mismatched- p22^phox^ 5'-TAG CAT AGC CCT CCG CTG GGG-3'. This ODN was annealed for 2 h while the temperature was lowered from 80℃ to 25℃ and then incubated with T4 ligase (Roche Diagnostic, GmbH, Mannheim, Germany) for 24 h at 16℃ to generate covalently ligated-decoy molecules. The ring formation of ODNs was examined on a 20% denaturing polyacrylamide gel. The VSMC were transiently transfected by Effectene® agent (QIAGEN Inc., CA, USA) according to the manufacturer's instructions.

.

**Table S1. RT-PCR primer sequences**

| ***Gene*** | ***5′ sequence*** | ***3′ sequence*** | ***bp*** | **GenBank Accession Number** | |
| --- | --- | --- | --- | --- | --- |
| *hCRP* | TGAATTCAGGCCCTTGTATC | TCCCAGCATAGTTAACGAGC | 935 | | NM_000567 |
| *hRef-1* | AAACCTCACCCAGTGGCAAA | AATTCAGCCACAATCACCCG | 242 | | S43127.1 |
| *rRef-1* | TGTATGAGGACCCTCCAGAT | GTCTCTTGGAGGCACAAGAT | 160 | | NM_024148.1 |
| *hp22^phox^* | TGGTACTTTGGTGCCTACTC | GCGGTCATGTACTTCTG | 119 | | NM_000101.3 |
| *hNOX2* | CGTCTTCCTCTTTGTCTGGT | CGACAGACTGGCAAGAGAAT | 153 | | NM_000397 |
| *hNOX4* | ACCTCAACTGCAGCCTTATC | CCTCATCTCGGTATCTTGCT | 254 | | NM_016931 |
| *hGADD153* | GTCTAAGGCACTGAGCGTAT | GACAGTGTCCGAAGAGAAAG | 203 | | S40706 |
| *hBax* | CAGAGTTCCAGACCATGTTG | CAGTGATGCAGCATGAAGTC | 230 | | AF520590.1 |
| *rBax* | AACAGTGTCTTGGGTCAGGT | CGCTCTTAAATAGGCTGGAG | 163 | | NM_053812 |
| *hBak* | AAGAAGCTGAGCGAGTGTCT | CTCACTCACCATCTGGAAGA | 449 | | NM_001291428 |
| *rBak* | GGTGGTTGCCCTTTTCTACT | CAAAGATGGTCACTGTCTGC | 203 | | NM_017059 |
| *hBcl2* | ACCGGGAGATAGTGATGAAG | CTCAAAGAAGGCCACAATCC | 322 | | NM_000633 |
| *rBcl2* | GTATGATAACCGGGAGATCG | CTGACTGGACATCTCTGCAA | 322 | | L14680.1 |
| *hp53* | CCTCAGCATCTTATCCGAGT | TGGAGTCTTCCAGTGTGATG | 211 | | AB082923 |
| *hMDM2* | ACCTCACAGATTCCAGCTTC | TACCTGAGTCCGATGATTCC | 313 | | NM_002392 |
| *h&rGAPDH* | GACCCCTTCATTGACCTC | GCTAAGCAGTTGGTGGTG | 374 | | BC083511.1 |

**Table S2. Primary antibodies for IP, IB, or IF**

| ***Name*** | ***Source/Purification*** | ***Catalog No.*** | | ***MW (kDa)*** | | **Application** | |
| --- | --- | --- | --- | --- | --- | --- | --- |
| *hCRP* | Rabbit polyclonal CRP (H90)  Mouse monoclonal CRP (CRP-8) | | Sc-30047, C 1688  (SANTA Cruz Biotech. USA)  (Sigma Aldrich, USA) | | 24-30 | | IP, IB, IF |
| *r&h p22^phox^* | Rabbit polyclonal CYBA (FL-195)  Mouse monoclonal CYBA(E-8) | | Sc-20781, Sc-271262  (SANTA Cruz Biotech. USA) | | 22 | | IB |
| *r&hRef-1* | Mouse monoclonal APEX1(C-4)  Goat polyclonal APEX1(E-17) | | Sc-17774, Sc-9919  (SANTA Cruz Biotech. USA) | | 37 | | IP, IB, IF |
| *hα-smooth*  *Muscle actin* | Rabbit polyclonal SMA  Rabbit polyclonal SMA | | ab5694 #14968  (abcam, UK; cell signaling, USA) | | 42 | | IF |
| *r&hSTAT3* | Rabbit polyclonal STAT3  Mouse monoclonal STAT3(124H6) | | #9132, #9139  (Cell Signaling, USA) | | 79, 86 | | IB |
| *r&hSTAT3-p* | Rabbit monoclonal p-STAT3(D3A7) | | #9145  (Cell Signaling, USA) | | 203 | | IB |
| *r&h JAK sampler* | Rabbit polyclonal JAK1&2 | | #9945  (Cell Signaling, USA) | | 125 | | IB |
| *p-JAK1&2* | Rabbit polyclonal JAK1&2-p | | #3771  (Cell Signaling, USA) | | 125 | | IB |
| *r&h Bax* | Mouse monoclonal Bax (B-9) | | Sc-7480  (SANTA Cruz Biotech. USA) | | 25 | | IB |
| *r&h Bak* | Rabbit polyclonal Bak | | #3814  (Cell Signaling, USA) | | 25 | | IB |
| *r&h Bcl2* | Mouse monoclonal Bcl2 (C-2) | | Sc-7382  (SANTA Cruz Biotech. USA) | | 28 | | IB |
| *p-Tyr* | Mouse monoclonal p-Tyr (PY99) | | Sc-7020  (SANTA Cruz Biotech. USA) | |  | | IP |
| *c-myc* | Rabbit polyclonal  Goat monoclonal c-Myc (9E10) | | A190-105A, #631206 (BETHYL Lab, INC USA)  (Clontech, USA) | | 322 | | IP, IB |
| *r&h p53* | Mouse monoclonal p53 (1C12)  Rabbit polyclonal p53 | | #2524, #9282  (Cell Signaling, USA) | | 53 | | IB, IF |
| *rCytochrome c* | Mouse monoclonal Cytochrome c  (6H2.B4) | | #556432 (BD Biosciences, USA) | | 15 | | IB |
| *r&h Caspase3* | Rabbit polyclonal Cas3  Rabbit polyclonal cleaved Cas3 (Asp175) | | #9662, #9661  (Cell Signaling, USA) | | 35, 17 | | IB |
| *p-JAK1&2* | Rabbit polyclonal JAK1&2-p | | #3771  (Cell Signaling, USA) | | 125 | | IB |
| *r&h α-tubulin* | Rabbit monoclonal tubulin (11H10) | | #2125  (Cell Signaling, USA) | | 52 | | IB |
| *r&h ubiquitin& monoubiquitin Ab* | Mouse monoclonal Ubquitin (FK2)  Rabbit polyclonal monoubiquitin(H2A) | | #04-263, ABE569  (EMD Millipore, USA)  (Sigma-Aldrich, USA) | | 64,22,14 | | IP, IB |
| *r&h COX IV* | Rabbit monoclonal COXIV (3E11) | | #4850  (Cell Signaling, USA) | | 17 | | IB |
| *r&h eIF6* | Rabbit monoclonal eLF6 (D16E9) XP | | #3833  (Cell Signaling, USA) | | 27 | | IB |


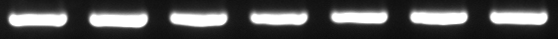

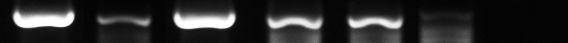


**CON**

**#5**

**#17**

**#28**

**#31**

**#35**

**#37**

**p22^phox^**

**GAPDH**


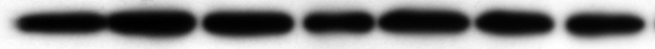

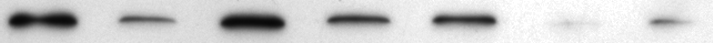


**CON**

**#5**

**#17**

**#28**

**#31**

**#35**

**#37**

**p22^phox^ Ab**

**α Actin**

**A**

**B**

**Figure S1. The mRNA and protein of p22^phox^ are knocked down in VSMCs with a lentiviral shp22^phox^-U6 vector system.**

After lentiviral shp22^phox^-U6 infection, mRNA and protein expression were observed in stable p22^phox^-knock down VSMC (p22^phox^KD) cultured after isolation as one cell line. It was confirmed by RT-PCR (A) and western blotting (B) that p22phox expression was knocked down in the 35th stable cell line. GAPDH was used as a representative amount of mRNA for loading control. α actin was used as a representative amount of protein for loading control.

**A**

**B**


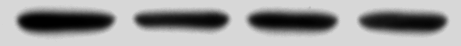

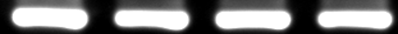

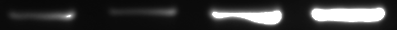

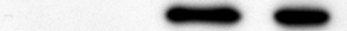


**tCRP**

**tMOCK**

**tCRP**

**tMOCK**

**GAPDH**

**α Actin**

**hCRP**

**CRP Ab**

**Figure S2. The mRNA and protein of human CRP are overexpressed in rat vascular smooth muscle cells (A10) through pcDNA3.1-human CRP cDNA transfection (tCRP).**

After pcDNA3.1 control vector and pcDNA3.1-human CRP cDNA vector were transfected into A10 cells, mRNA and protein expression were observed in tMOCK- and tCRP- A10 cells. CRP overexpression was confirmed by RT-PCR (A) and western blotting (B). GAPDH was used as a representative amount of mRNA for loading control. α actin was used as a representative amount of protein for loading control.

**A**


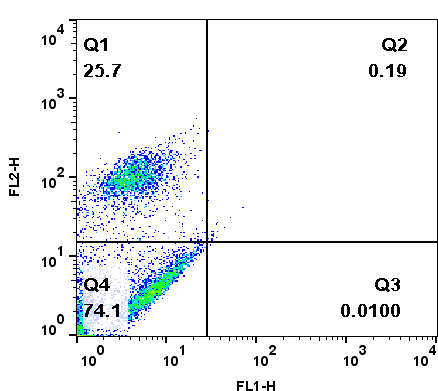

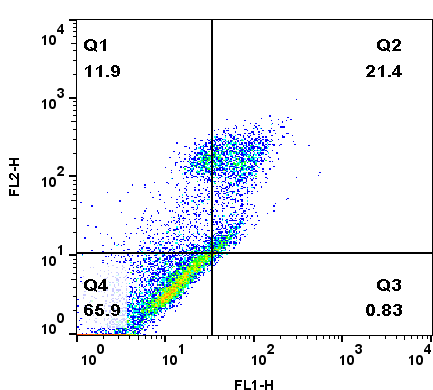


**MOCK**

**tCRP**

**B**

**Figure S3. Overexpressed CRP induces Apoptosis**

5 x 10^5^ rat VSMCs (A10) were transfected with pcDNA3.1 (MOCK) or pcDNA3.1-human CRP cDNA (tCRP) expression vector for 24 hours. (A) Apoptosis and cell death of CRP overexpressing cells were obtained using Annexin V-FITC and propidium iodide using an apoptosis detection kit. (B) The mean values of the specific cell-associated fluorescence are shown with bar graph.

**MOCK**


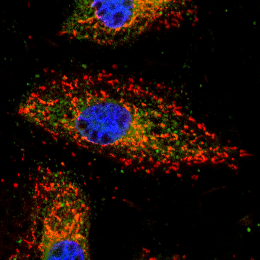

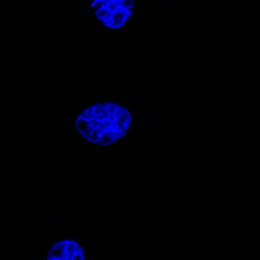

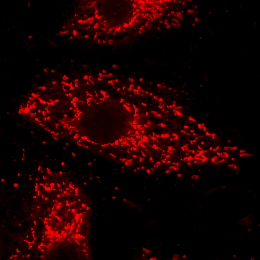

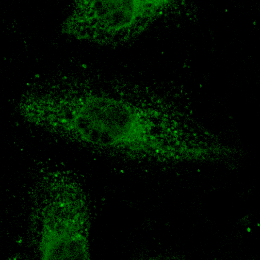

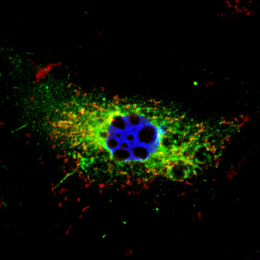

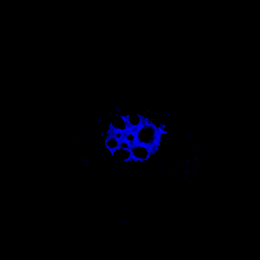

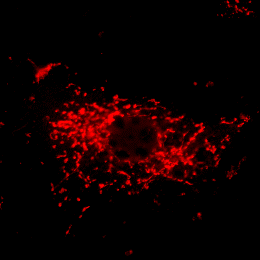

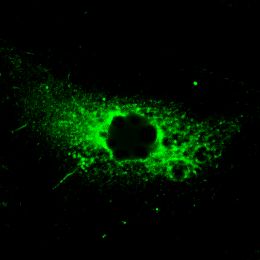


**tRef-1**

**a. DAPI**

**b. Ref-1 Ab-FITC**

**c. Mitochondria tracker**

**d. a+b+c**

**i. DAPI**

**j. Ref-1 Ab-FITC**

**k. Mitochondria tracker**

**l. i+j+k**


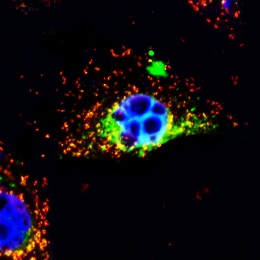

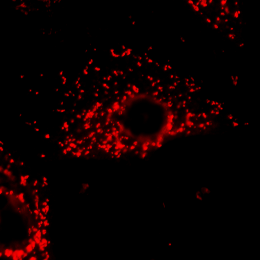

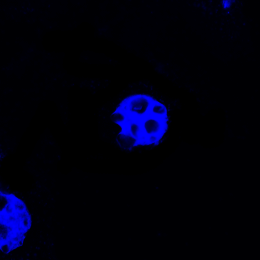

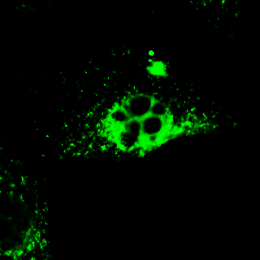


**e. DAPI**

**f. Ref-1 Ab-FITC**

**g. Mitochondria tracker**

**h. e+f+g**

**tCRP + tRef-1**

**Figure S4. Overexpressed Ref-1 with CRP is co-localized or closely adjacent to mitochondria**

A10 cells were transfected with the pcDNA3.1 vector (tMOCK), pcDNA3.1-human CRP (tCRP), or pcDNA3.1-human Ref-1 cDNA (tRef-1) for 48 h. The total level of the Ref-1 proteins and localization of mitochondria in A10 cells were analysed through immunofluorescence (IF) using MitoTracker (MitoTracker™ Red CMXRos, M7512), goat-anti Ref-1 antibody, and FITC-conjugated secondary anti-goat antibody. The nuclei were stained with 4',6-diamidino-2-phenylindole (DAPI). Magnification, 630 X. CRP expression pattern and mitochondria localization are shown using confocal microscopic images (panels a, e, and i: nucleus is blue, panels b, f, and j: Ref-1 is green at 488 nm, panels c, g, and k: mitochondria are red at 578 nm).


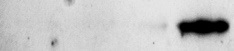


**CRP**

**IP : Myc**

**total**

**lysate**

**Myc-tRef-1**

**CRP**

**β-actin**

**−**

**−**

**+**

**+**

**tCRP**

**+**

**−**

**+**

**−**

**Ref-1**


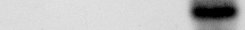


**IP: Myc**

**total**

**lysate**


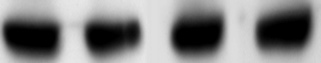


**Myc-tCRP**


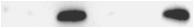


**CRP**

**CRP**

**β-actin**

**IgG**

**CRP**

**+**

**−**

**+**

**−**

**A**

**B**

**Figure S5. Overexpressed CRP binds to Ref-1.**

A10 cells were transfected with pEF1α-myc vector (myc-tMOCK) and pEF1α-myc-hCRP cDNA (myc-tCRP) for 48 hours. The total level of CRP protein was detected by mouse-anti CRP antibody (A). A10 cells were transfected with pEF1α-myc vector (myc-tMOCK), pcDNA3.1-CRP cDNA (tCRP), and pEF1α-myc-hCRP cDNA (myc-tCRP) for 48 hours. The total levels of the CRP and Ref-1 proteins were detected by mouse-anti CRP and mouse-anti Ref-1 antibodies (B). Total lysates of A and B cells were cross-linked and immunoprecipitated by rabbit-anti myc antibody and analyzed for CRP expression by mouse-anti CRP antibody. α-actin was used as a representative amount of protein for loading control.

**c. CRP Ab-Alexa Flour 680 680**


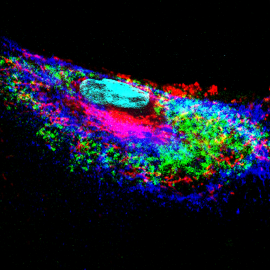

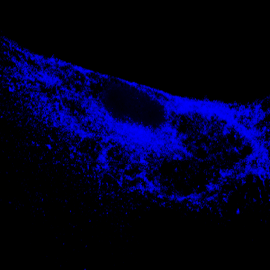

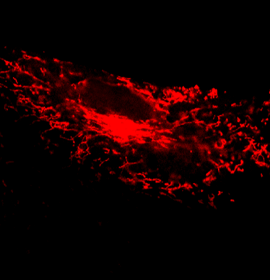

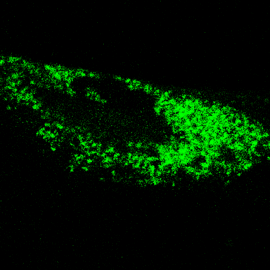

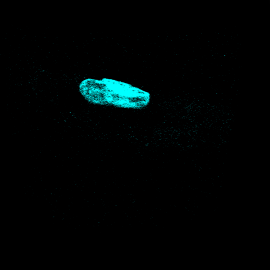

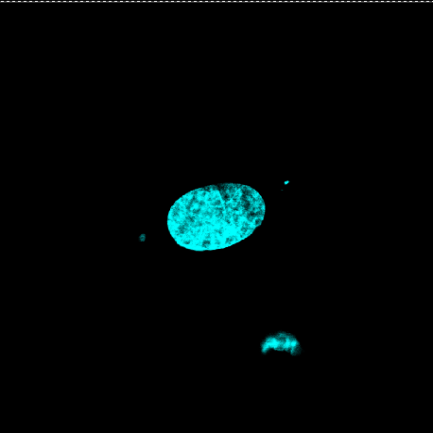

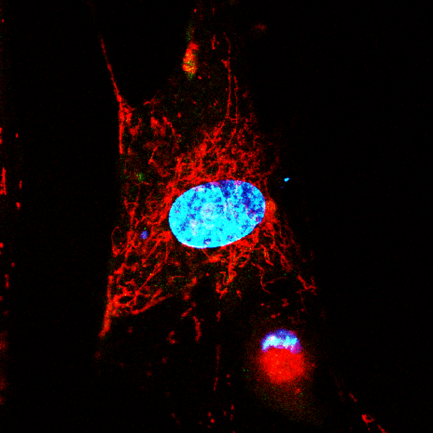

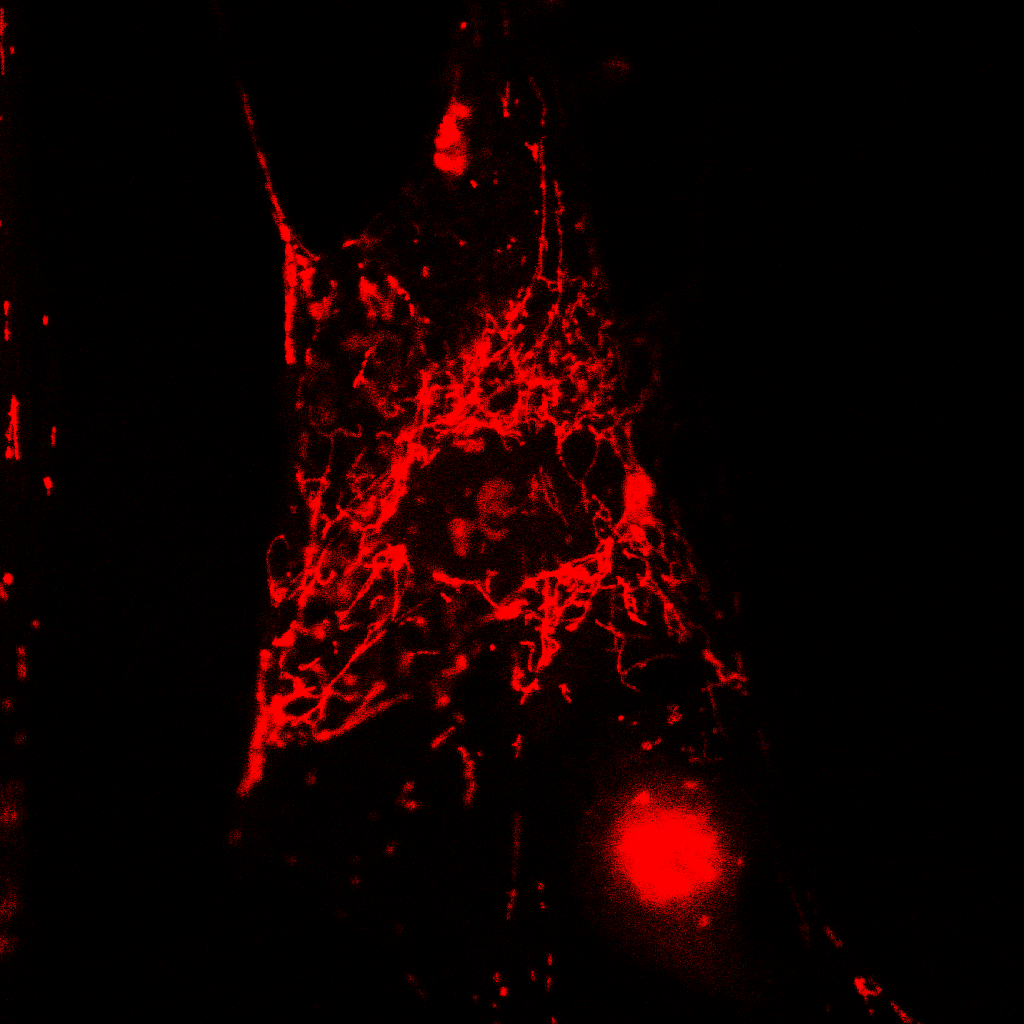

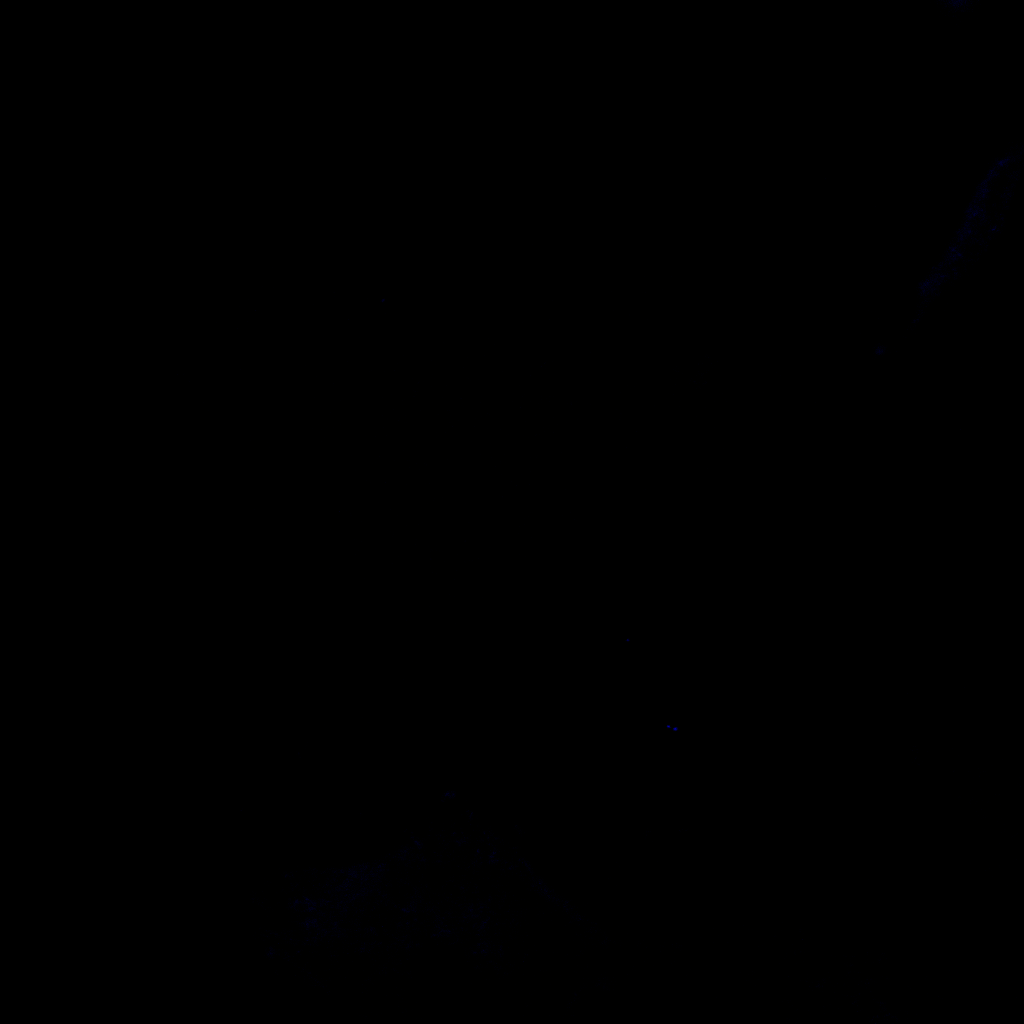

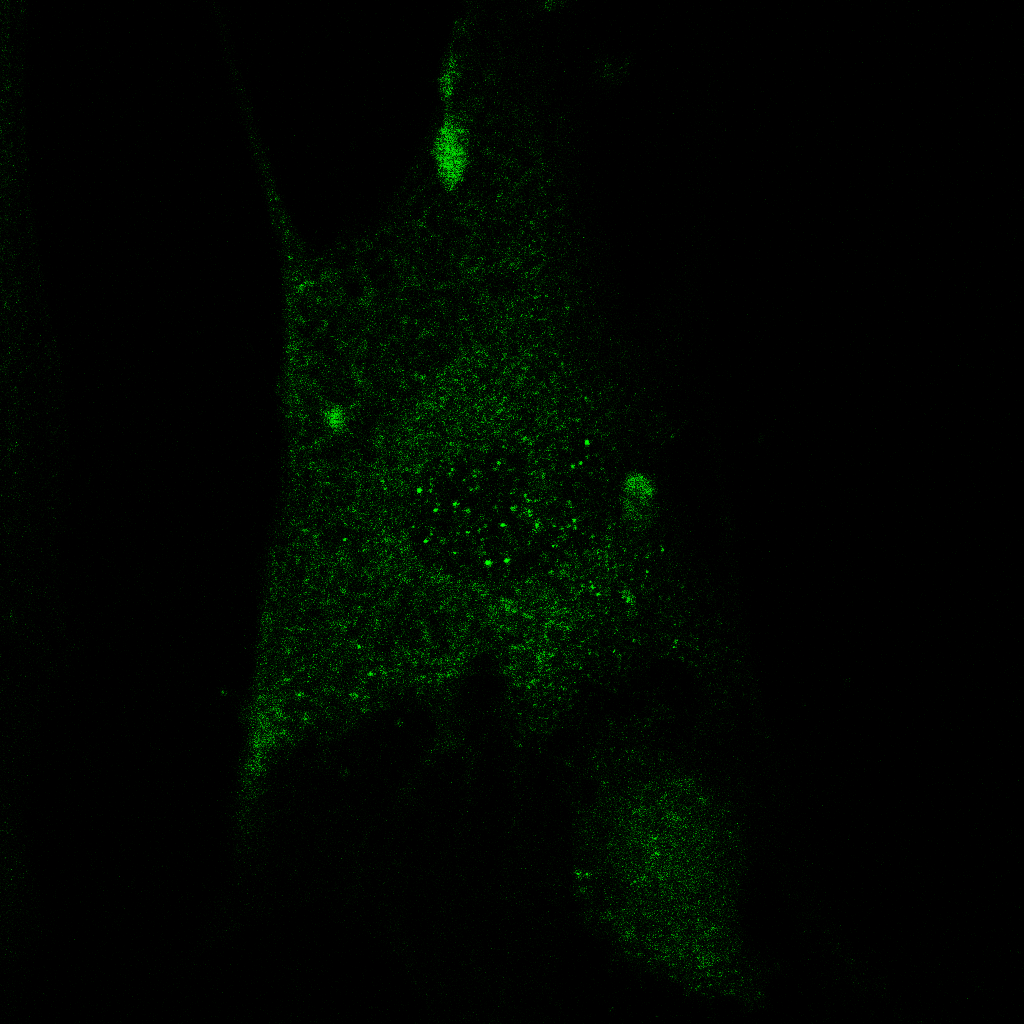


**a. DAPI**

**e. a+b+c+d**

**MOCK**

**tCRP**

**f. DAPI**

**g. p53-FITC**

**h. CRP Ab-Alexa Flour 680 680**

**j. f+g+h+i**

**b. p53 Ab-FITC**

**d. Mitochondria**

**c. CRP Ab-Alexa Flour 680 680**

**i. Mitochondria**

**Figure S6. Overexpressed CRP induces p53 expression and co-localizes with p53 in mitochondria and cytoplasm of VSMCs.**

A10 cells were transfected with the pcDNA3.1 vector (tMOCK) and pcDNA3.1-human CRP (tCRP) for 48 h. CRP and p53 proteins in A10 cells were detected using rabbit anti-CRP, Alexa Fluor® conjugated anti-rabbit secondary antibody, mouse anti-p53 antibody, and FITC-conjugated anti-mouse secondary antibody. Mitochondrial localization was detected using MitoTracker™ Red CMXRos. The total expression levels of CRP and p53 were analysed through IF. Nuclei were stained with DAPI. Magnification, 630X. CRP and p53 expression patterns and mitochondria localization are shown using confocal microscopic images (panels a and f: the nucleus is blue, panel b and g: p53 is FITC green, panel c and h: CRP is blue at 680 nm, and panels d and i: mitochondria are red at 578–599 nm).

**A**

**p22^phox^**

**CRP**

**GAPDH**

**CRP-pcDNA3.1 – + +**

**p22^phox^ decoy – – +**


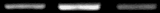

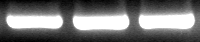

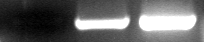


**MOCK**


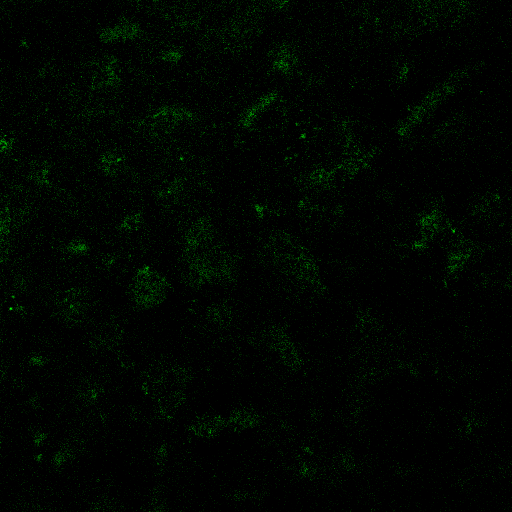

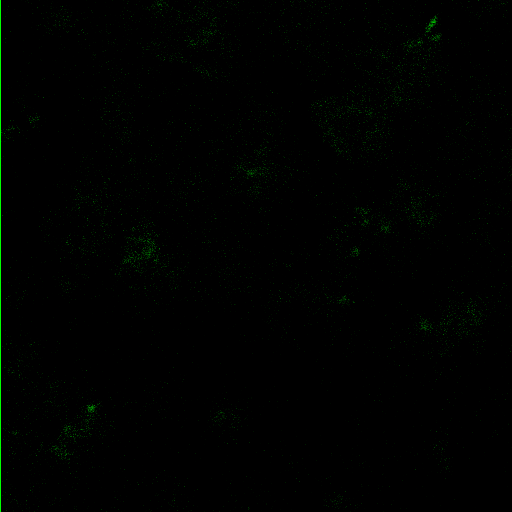

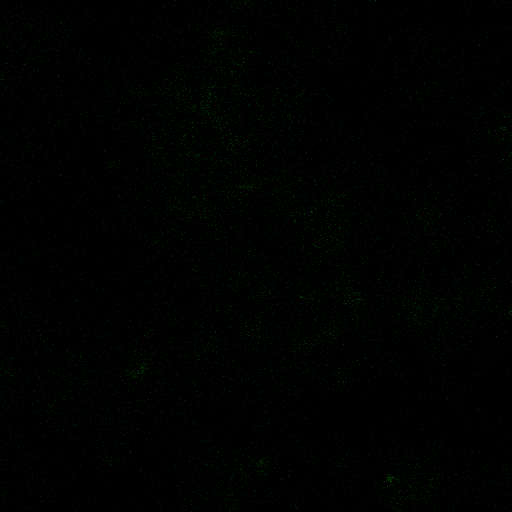

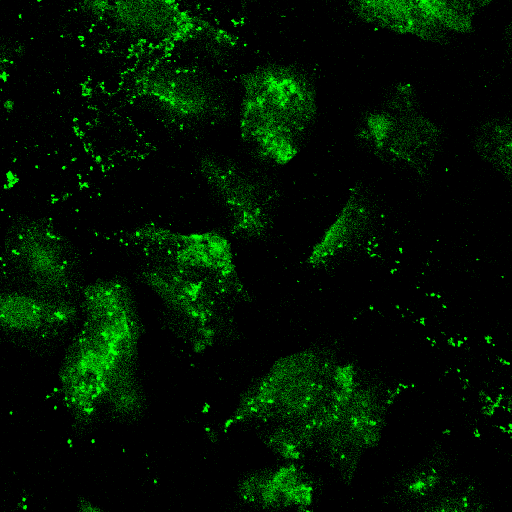

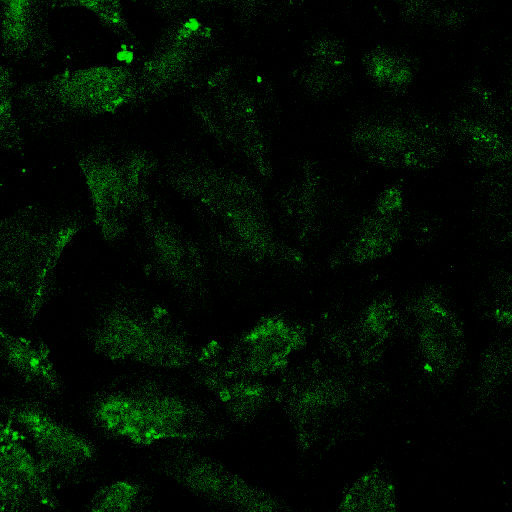

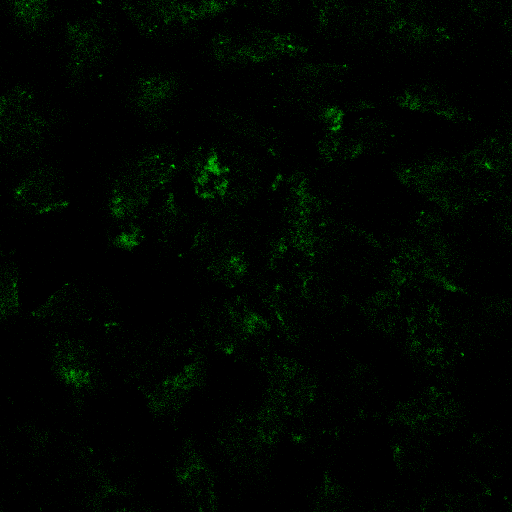


**-ODNs**

**tCRP**

**-ODNs**

**10nM**

**50nM**

**10nM**

**50nM**

**+ ODNs of p22^phox^**

**+ ODNs of p22^phox^**

**B**

**Figure S7. The p22^phox^ subunit of NOXs is suppressed with p22 ^phox^–specific ODNs.**

A10 cells were co-transfected with/without pcDNA3.1-human CRP cDNA and 10nM p22^phox^ specific ODNs or mismatched ODNs for suppression of NOXs. (A) Transcripts of CRP and p22^phox^ were produced by RT-PCR. The amount of total mRNA is shown with GAPDH as the house-keeping gene during the PCR cycles (27 cycles were performed). (B) human VSMCs were treated as described in Panel A. To determine whether CRP-activated VSMCs suppressed expression of p22^phox^ protein, cells were stained with anti-p22^phox^ antibody and FITC-conjugated secondary antibody. Confocal microscopy images are shown with green color fluorescence for the p22^phox^ proteins.

**0**

**Figure S8. Analysis of VSMCs DNA synthesis by BrdU staining.**

Cultured human VSMC were transfected with tMock, tCRP, tRef-1 or tCRP/tRef-1 for 24 hours. The transfected cells were then permeabilized and incubated with anti-BrdU primary antibody. Cell-associated BrdU antibody was detected using Alex fluor plus 555-conjugated secondary antibody, then measured by a VICTOR3™ Multilabel Plate Reader (PerkinElmer, Inc., Wallac Oy, Turku, Finland). (** p < 0.01 significant, ***; p < 0.001 significant)
