# Supplementary material for: Radiation‐induced C‐reactive protein triggers apoptosis of vascular smooth muscle cells through ROS interfering with the STAT3/Ref‐1 complex
Source: J Cell Mol Med. 2022 Feb 17;26(7):2104–18. doi: 10.1111/jcmm.17233 (PMC8980952; doi:10.1111/jcmm.17233)
Supplement: Supplementary file 2 — Supplementary Material [file JCMM-26-2104-s003.docx]

***Comment 1 (Details)***

***Figure No. Figure 1E***

**E**

**0 2 4 8**

**(Gy/24h)**

**GADD153**

**Bcl2**

**Bax**

**GAPDH**

**Ref1**

**CRP**

**p22^phox^**

**NOX4**


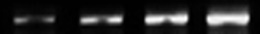

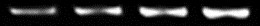

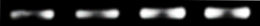

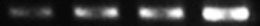

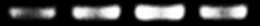

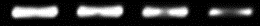

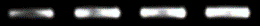

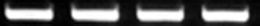


**Original Image**


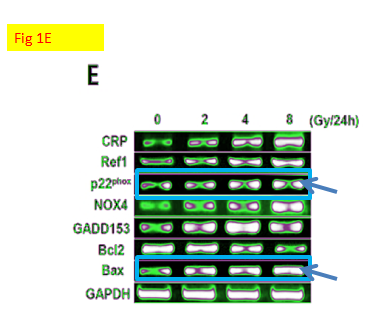


**Analysed Image**

***Adjustments in Photoshop (brightness/contrast, and curves) were used to analyze the image. The analysis indicates that the background of the bands 'p22phox and Bax' lanes is very clear (and not smudgy; as indicated in blue boxes and arrows), which suggests that the bands might have been placed into a standard background. Hence, the authors should be requested to provide an explanation and raw data (original gel blots) for verification.***

***Comment 1 (Author Request).***

***The authors should be requested to provide an explanation and raw data (original gel blots) for verification.***

**Response 1:**.

Regarding Bax, there is original data in the experimental notes dated February 8, 2021. In this experiment note, 4 original bands are indicated in red text (Response 1-1). The following picture is the original image film related to Bax bands. It was obtained from the Gel Doc equipment. In the experimental note dated February 8, 2021, the first 4 of the second row are the original Bax bands (Response 1-2).

Regarding P22^phox^, there is original data in the experimental note dated February 15, 2021. In the experimental note, 4 original bands are indicated in red text (Response 1-3). The following picture is the original image film related to p22^phox^ bands. It was obtained from the Gel Doc equipment. As in the experimental note dated February 15, 2021, the first 4 of the second row are the original p22^phox^ bands (Response 1-4).

When the original data was acquired, the background of the print state was too dark. To express clear bands like figure 1E submitted just before, the data was expressed by adjusting the contrast of white/black balance. However, no movement or band changes were artificially adjusted.

And based on these original Gel Doc images of p22^phox^ and Bax, a new figure 1E (Response 1-5) has been created and reflected by replacing the previous figure.

We hope that you confirm and approve the figure replacement.


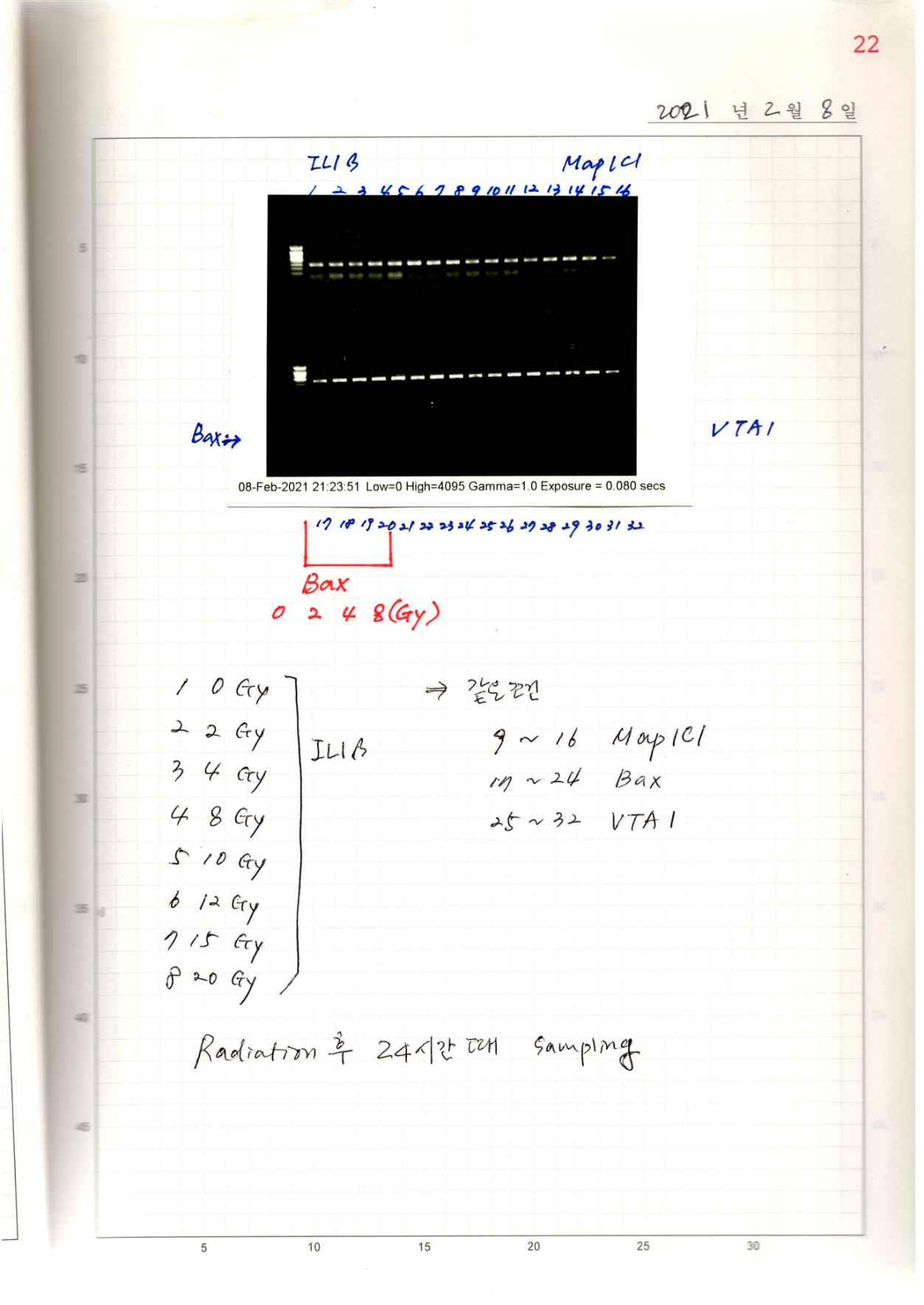


Response1-1. Experimental note for Bax band (Marked in Red)


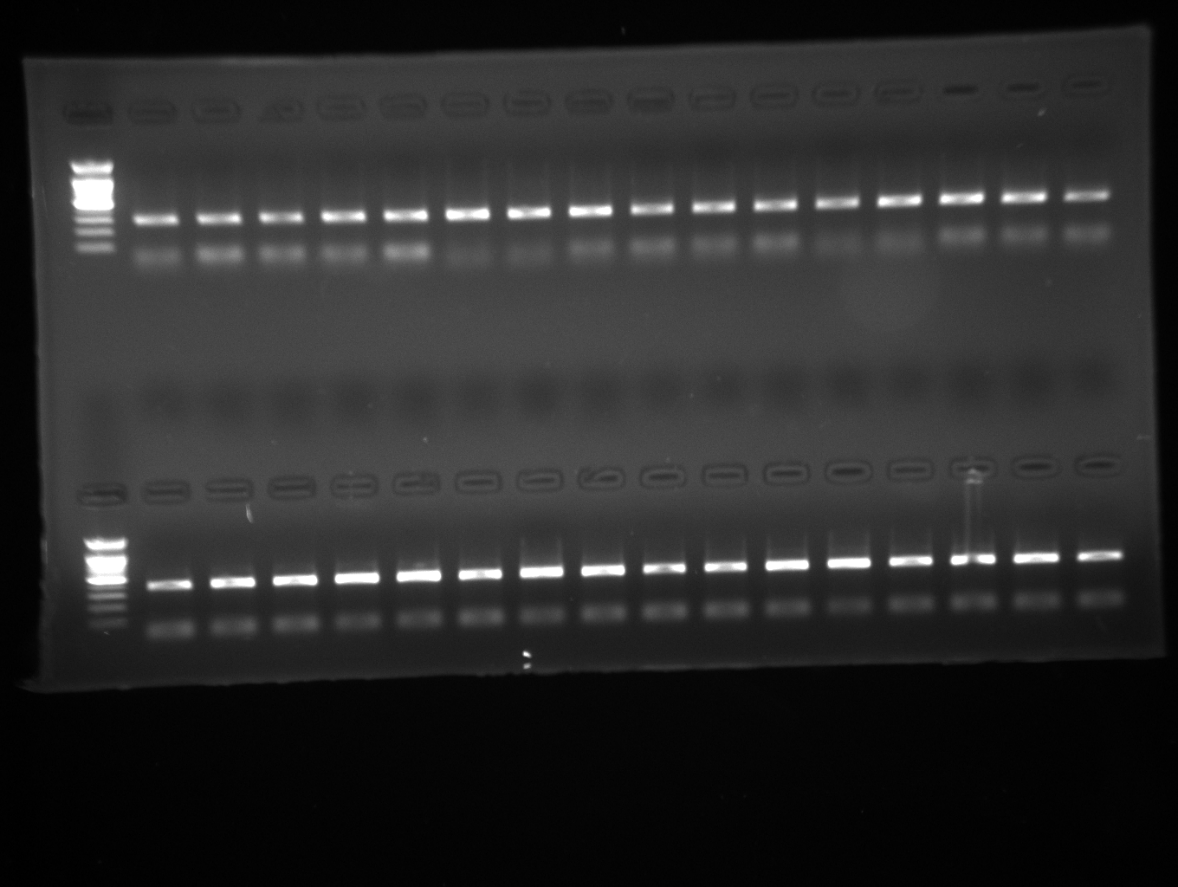


Response 1-2. Bax band images of Gel Doc


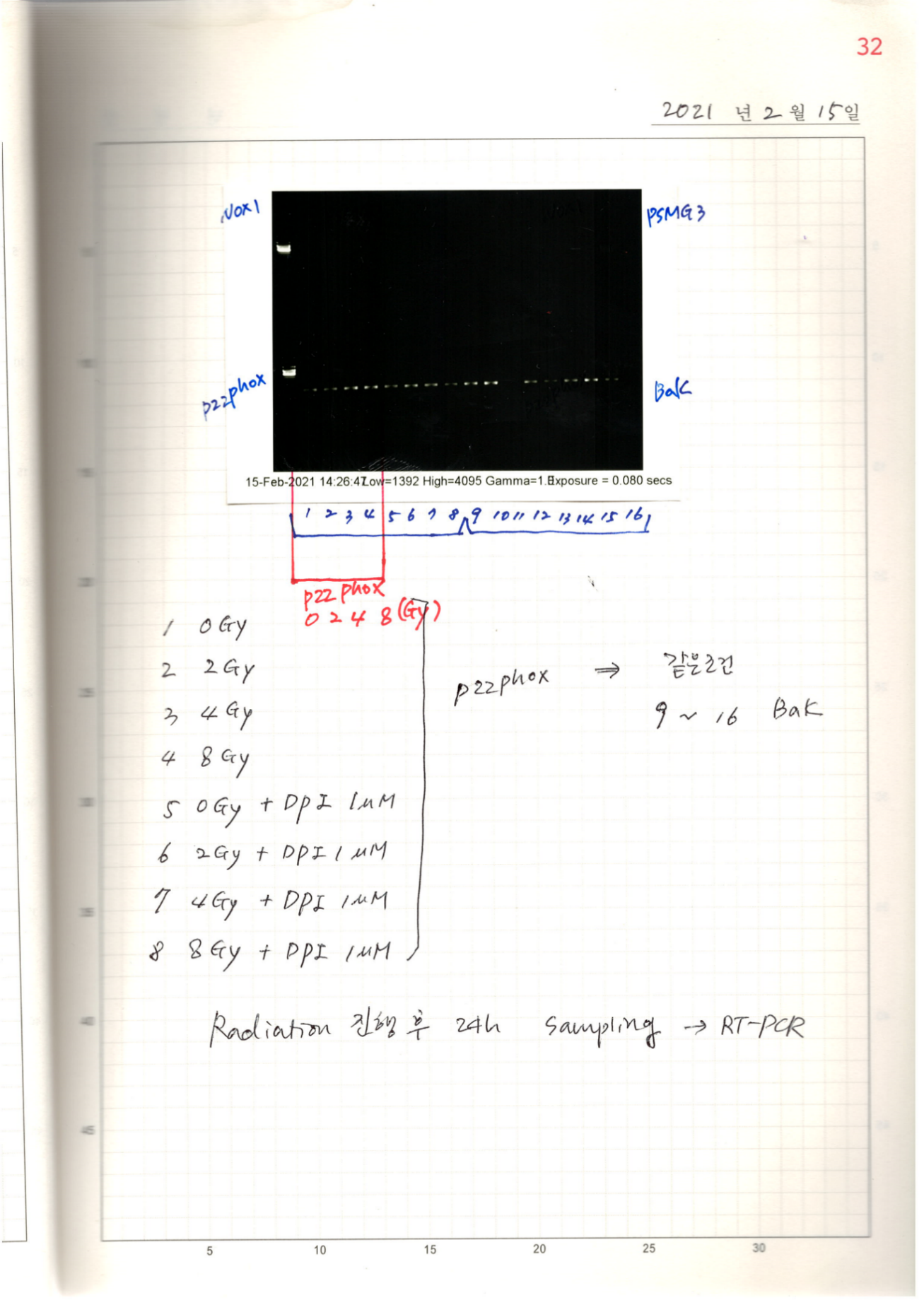


Response 1-3. Experimental note for p22^phox^ band (marked in Red)


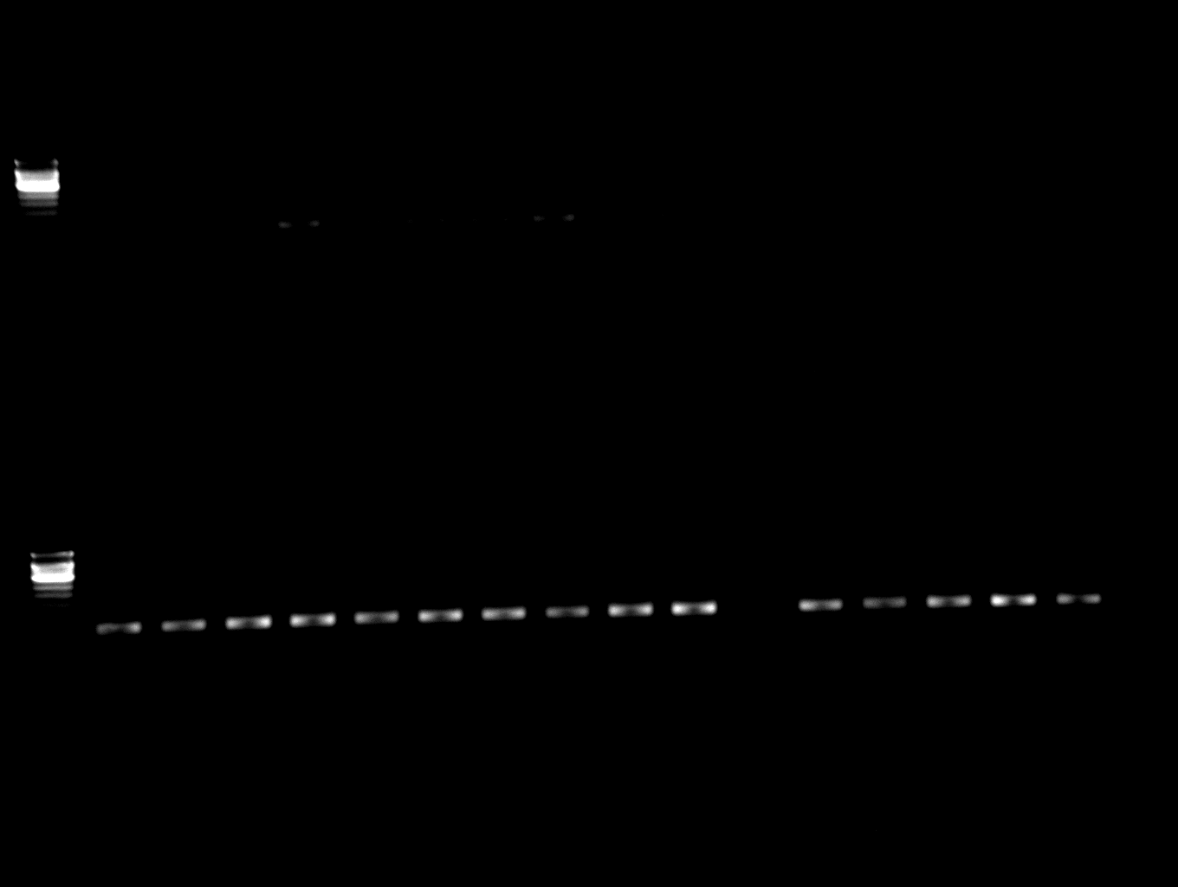


Response 1-4. P22^phox^ band image of Gel Doc (the first 4 of the band line)

Response 1-4. P22phox band image of Gel Doc (the first 4 of the band line)

**Fig 1E**

**0 2 4 8**

**(Gy/24h)**

**GADD153**

**Bcl2**

**Bax**

**GAPDH**

**Ref1**

**CRP**

**p22^phox^**

**NOX4**


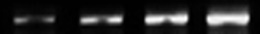

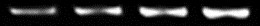

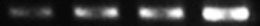

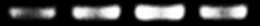

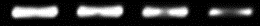

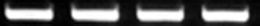

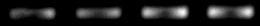

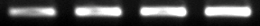


Response 1-5. New image of figure 1E
