# Supplementary material for: Radiation‐induced C‐reactive protein triggers apoptosis of vascular smooth muscle cells through ROS interfering with the STAT3/Ref‐1 complex
Source: J Cell Mol Med. 2022 Feb 17;26(7):2104–18. doi: 10.1111/jcmm.17233 (PMC8980952; doi:10.1111/jcmm.17233)
Supplement: Supplementary file 3 — Supplementary Material [file JCMM-26-2104-s004.docx]

***Comment 2 (Details)***

***Figure No. Figure 3D***

**D**

**hRef-1**

**hNOX2**

**hNOX4**

**p53**

**hMDM2**

**hGAPDH**

**hrCRP**

**_**

**+**


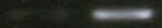

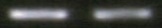

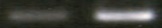

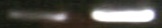

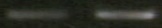

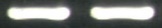

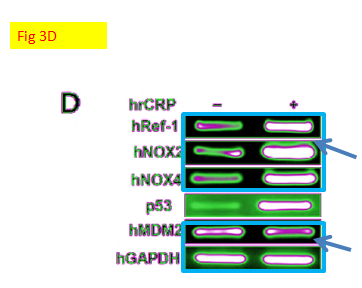


**Original Image**

**Analysed Image**

***Adjustments in Photoshop (brightness/contrast, and curves) were used to analyze the image. The analysis indicates that the background of the bands in most of the lanes is very clear (and not smudgy; as indicated in blue boxes and arrows), which suggests that the bands might have been placed into a standard background. Hence, the authors should be requested to provide an explanation and raw data (original gel blots) for verification.***

***Comment 2 (Author Request).***

***The authors should be requested to provide an explanation and raw data (original gel blots) for verification.***

**Response 2:**.

We are providing the experimental notes including original raw data. There are original Gel Doc images in the notes. The first experimental note dated July 22, 2009, has the original data of the experiment result confirming the amount of GAPDH expression (Response 2-1).

The following experimental note dated July 23, 2009, has the original data confirming the mRNA expression of hRef-1, hNOX2, hNOX4, p53, and hMDM2 (Response 2-2).

The band descriptions of experimental note (Response2-1 and Response 2-2) are presented in (Response 2-3).

And based on these original raw data images of hRef-1, hNOX2, hNOX4, hp53, hMDM2, and GAPDH, a new figure 3D (Response 2-4) has been created and reflected by replacing the previous figure. We hope that you confirm and approve the figure replacement.


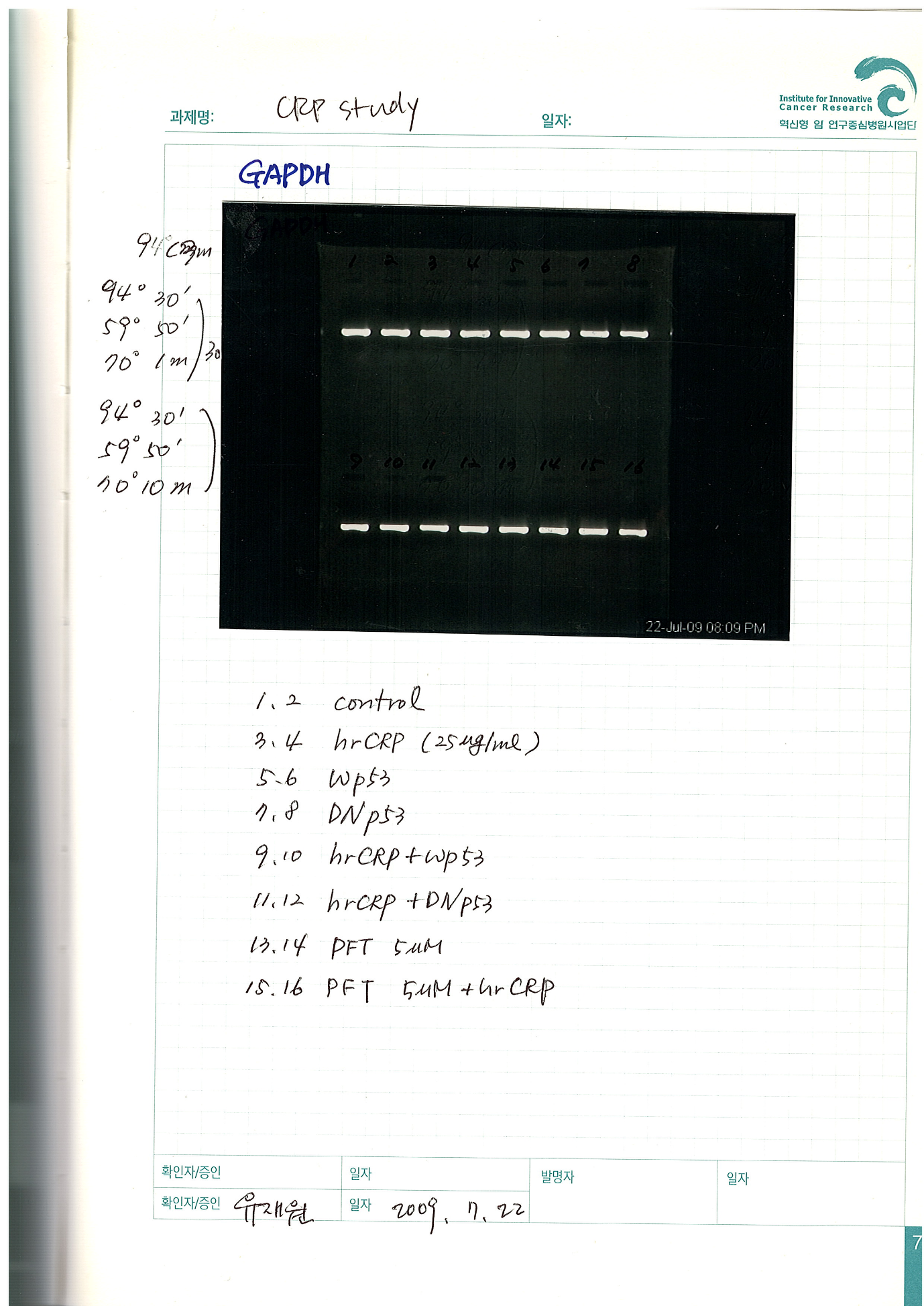


Response 2-1. Experimental note for GAPDH band (2^nd^ and 3^rd^ bands in the first line)

***
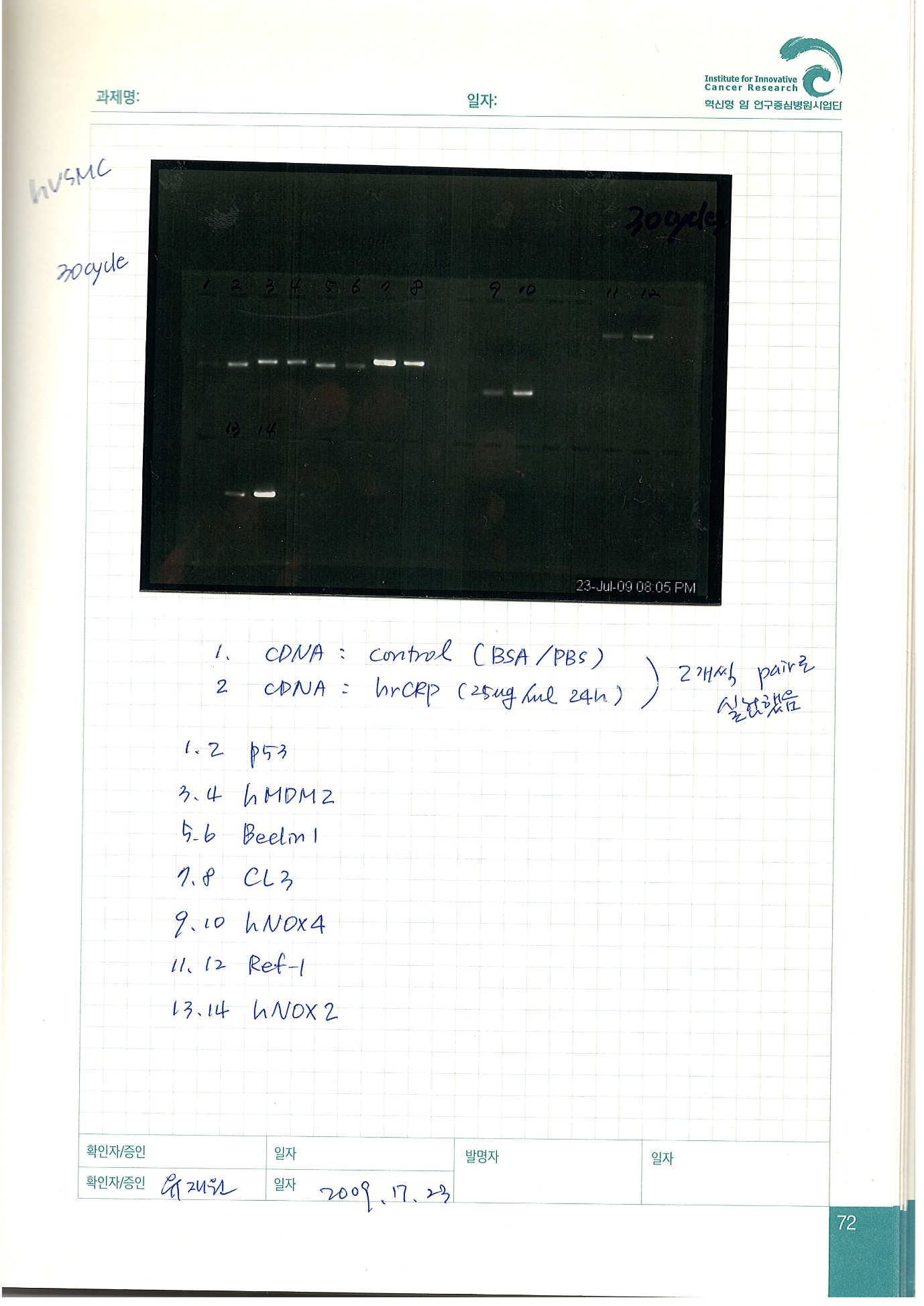
***

Response 2-2. Experimental note for hRef-1, hNox2, hNox4, p53, hMDM2 bands


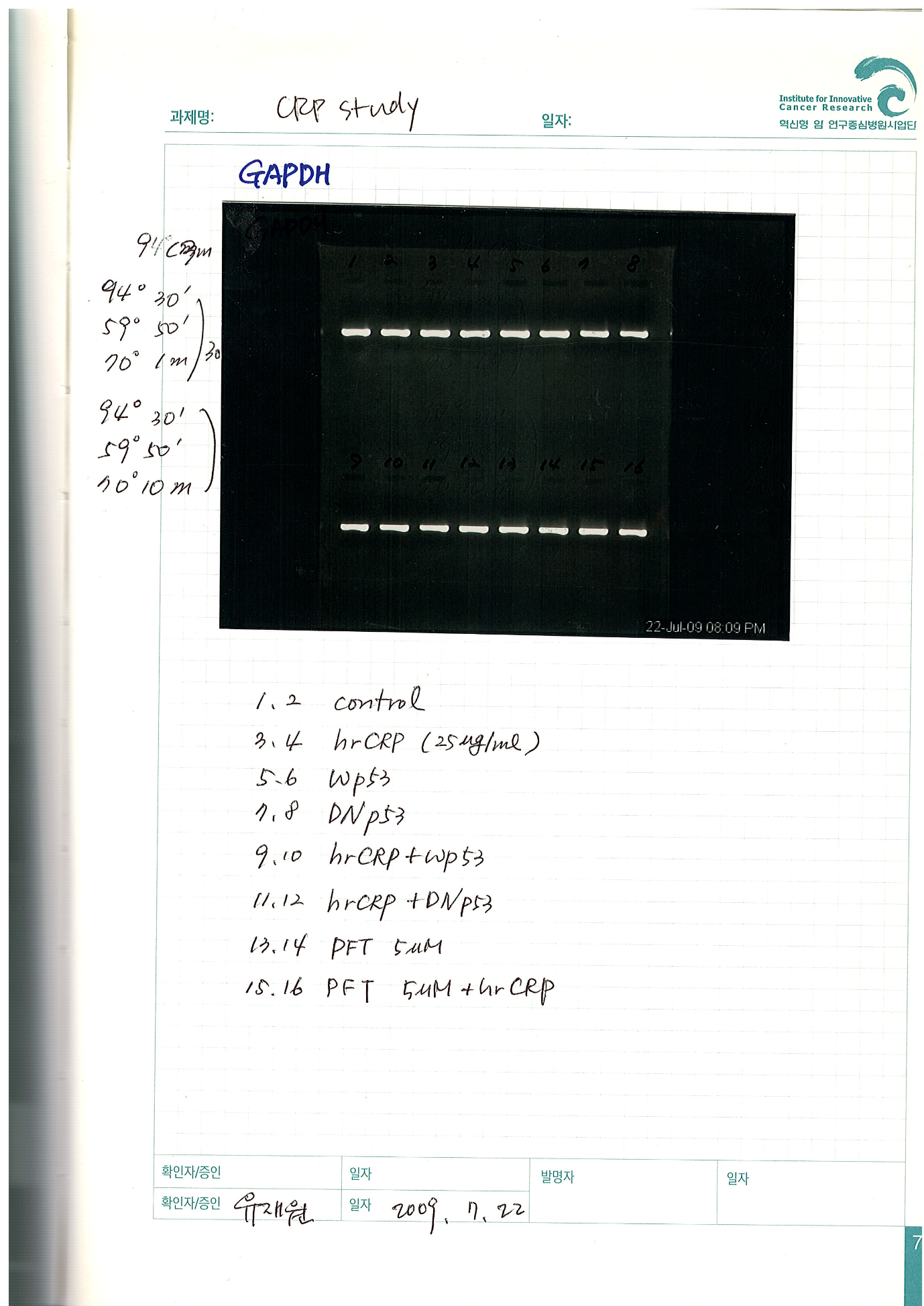


GAPDH


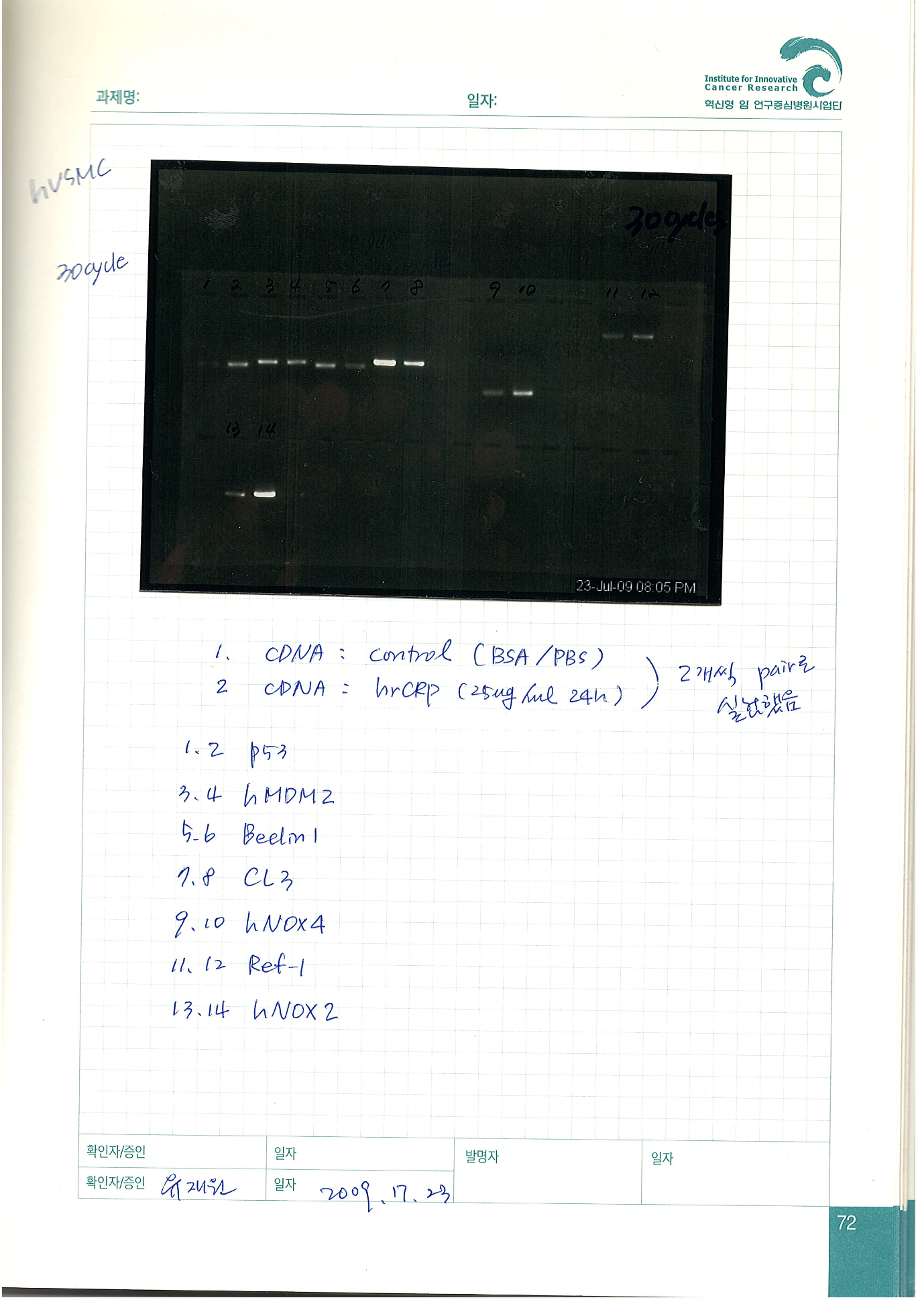


p53

hMDM2

hNOX4

hRef-1

hNOX2

Response 2-3. Description of band information in (Response 2-1) and (Response 2-2)

**Fig 3D**

**hRef-1**

**hNOX2**

**hNOX4**

**p53**

**hMDM2**

**hGAPDH**

**hrCRP - +**


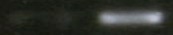

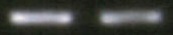

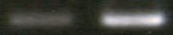

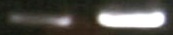

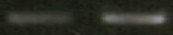

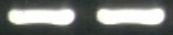


Response 2-4. The new image of figure 3D
