# Supplementary material for: Radiation‐induced C‐reactive protein triggers apoptosis of vascular smooth muscle cells through ROS interfering with the STAT3/Ref‐1 complex
Source: J Cell Mol Med. 2022 Feb 17;26(7):2104–18. doi: 10.1111/jcmm.17233 (PMC8980952; doi:10.1111/jcmm.17233)
Supplement: Supplementary file 4 — Supplementary Material [file JCMM-26-2104-s007.docx]

***Comment 3 (Details)***

***Figure No. Figure 5A***


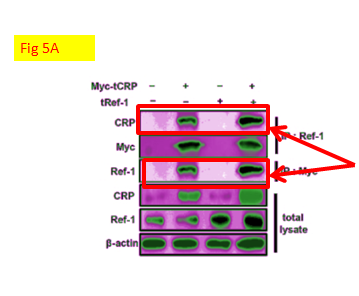


**CRP**

**IP : Ref-1**

**total**

**lysate**

**tRef-1**

**CRP**

**Myc**

**β-actin**

**−**

**−**

**+**

**+**

**Myc-tCRP**

**+**

**−**

**+**

**−**


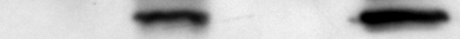

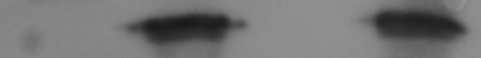


**Ref-1**

**IP : Myc**

**Ref-1**


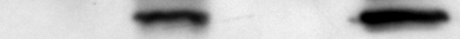


**Original Image**

**Analysed Image**

***Adjustments in Photoshop (change in brightness/contrast and curves) were used to analyze the image. The analysis indicates the presence of identical bands in 'CRP and Ref-1' lanes (highlighted using red boxes and arrows). Therefore, the authors should be requested to provide an explanation.***

***Comment 3 (Author Request).***

***The authors should be requested to provide an explanation for the duplication of the bands.***

**Response 3:**

The same experimental result image was duplicated in the process of preparing the manuscript submission due to our mistake. However, since it is an experimental result that has been proven through several experiments, the original data of the exact experimental result exists.

The original data films of the experimental results are in (Response 3-1). The results of this figure were an important part of proving the hypothesis of our studies. For self-confidence, the expression was confirmed several times through various experiments by cloning into 6 or more expression vectors. As shown in supplement figure 5B, on the contrary, by newly Ref-1 cDNA cloning in myc-tagging vector, it was confirmed and proved whether CRP and Ref-1 proteins form a complex with each other. To check whether the expression of each protein is clear, a simultaneous experiment with an IgG antibody was performed during immunoprecipitation to prove that it is not a non-specific band.


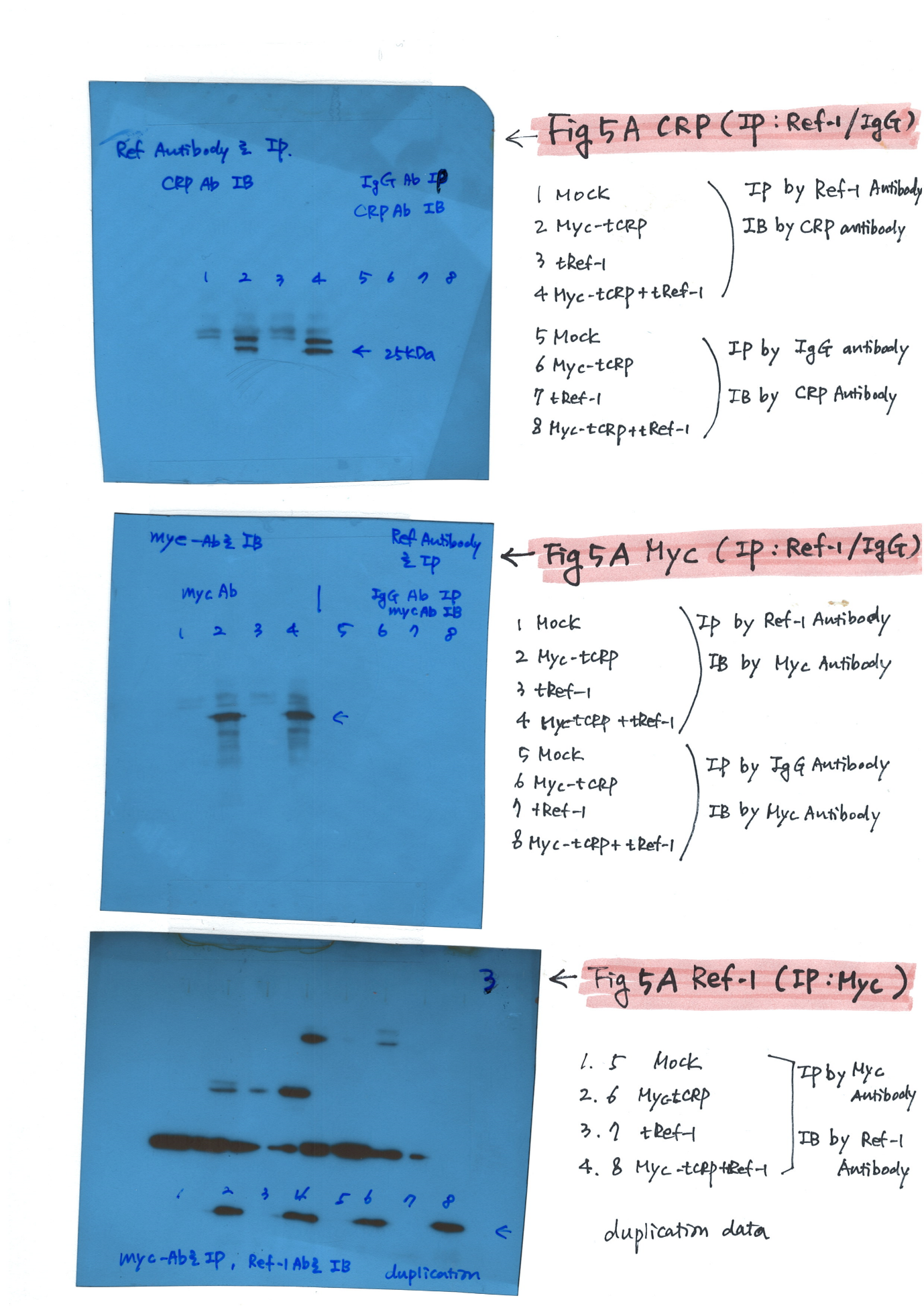


Response 3-1. Original film for CRP and Ref-1 expression

We have created the revised figure 5A (Response 3-2), reflecting the experimental result image we were originally trying to express. And we have reflected by replacing the previous figure. We hope that you confirm and approve the figure replacement.

Response 3-2. The new image of Figure 5A

**CRP**

**IP : Ref-1**

**total**

**lysate**

**tRef-1**

**CRP**

**Myc**

**β-actin**

**−**

**−**

**+**

**+**

**Myc-tCRP**

**+**

**−**

**+**

**−**


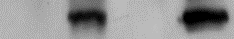

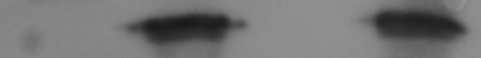


**Ref-1**

**IP : Myc**

**Ref-1**


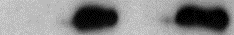


**Fig 5A**
