# Supplementary material for: Radiation‐induced C‐reactive protein triggers apoptosis of vascular smooth muscle cells through ROS interfering with the STAT3/Ref‐1 complex
Source: J Cell Mol Med. 2022 Feb 17;26(7):2104–18. doi: 10.1111/jcmm.17233 (PMC8980952; doi:10.1111/jcmm.17233)
Supplement: Supplementary file 5 — Supplementary Material [file JCMM-26-2104-s002.docx]

***Comment 4 (Details)***

***Figure No. Figure 6A***

**A**


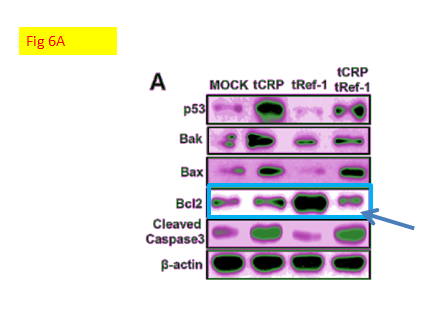


**MOCK**

**tCRP**

**tCRP**

**tRef-1**

**tRef-1**

**Bax**

**Cleaved**

**Caspase3**

**p53**

**β-actin**

**Bcl2**


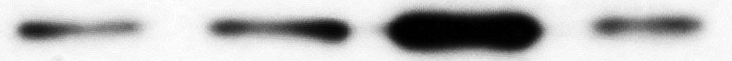

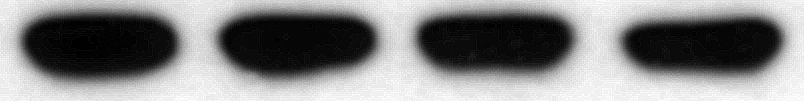

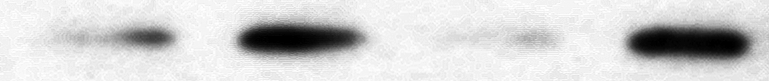

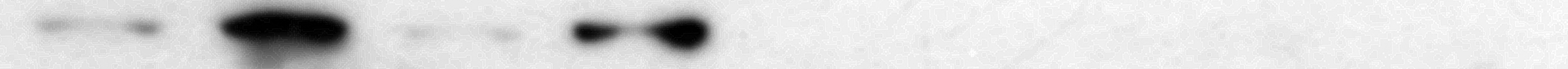

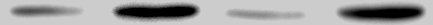


**Bak**

**Original Image**

**Analysed Image**

***Adjustments in Photoshop (change in levels and curves) were used to analyze the image. The analysis indicates that the background of the bands 'Bcl2' lane is very clear (and not smudgy; highlighted using a blue box). As this might have been caused due to high contrast, the authors should be requested to replace the gel blot with the original unmodified image.***

***Comment 4 (Author Request).***

***The authors should be requested to replace the gel blot with the original unmodified image.***

**Response 4:**

We are presenting original film image of the resulting data related to Bcl2 immunoblotting experiments (Response 4-1). In the film, 4 original bands are indicated in blue text.

When we got the result, the band pattern was so clear that we converted white/black image then adjusted the white brightness to better represent the experimental results. However, no other artificial modifications were performed on the original data.

And based on these original film image of Bcl2, a new figure 6A (Response 4-2) has been created and reflected by replacing the previous figure. We hope that you confirm and approve the figure replacement.


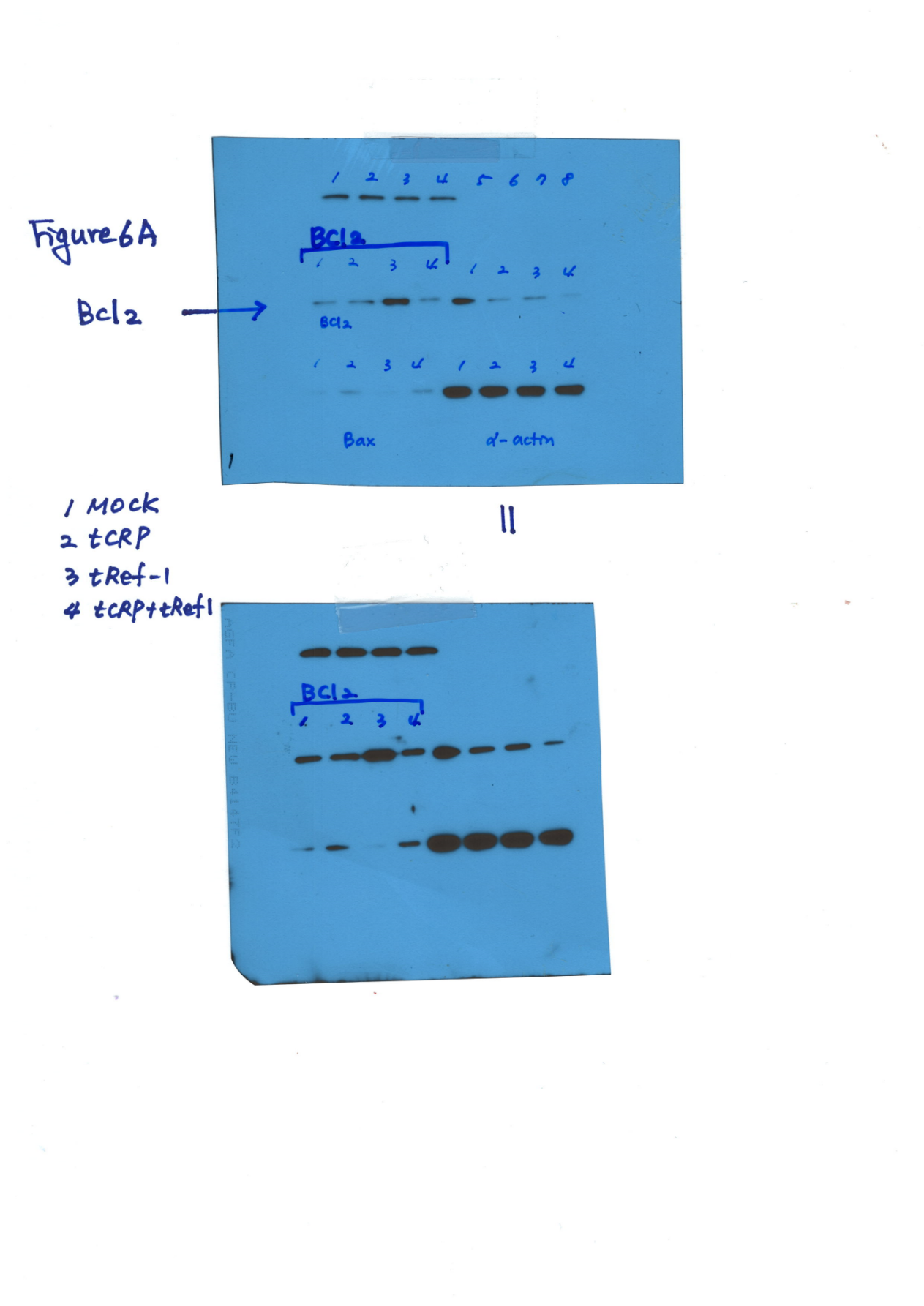


Response 4-1. Original film for Bcl2 expression (marked in blue text)

**MOCK**

**tCRP**

**tCRP**

**tRef-1**

**tRef-1**

**Bax**

**Cleaved**

**Caspase3**

**p53**

**β-actin**

**Bcl2**


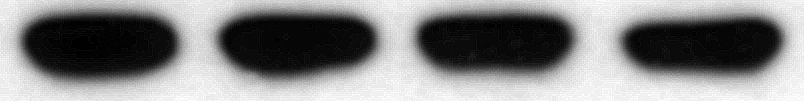

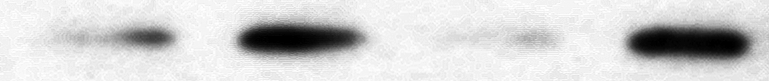

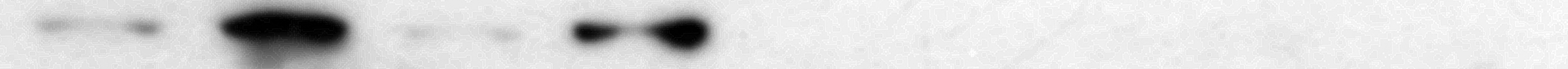

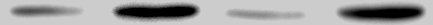


**Bak**

**Fig 6A**


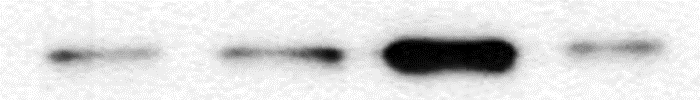


Response 4-2. The new image of Figure 6A
