# Supplementary material for: Radiation‐induced C‐reactive protein triggers apoptosis of vascular smooth muscle cells through ROS interfering with the STAT3/Ref‐1 complex
Source: J Cell Mol Med. 2022 Feb 17;26(7):2104–18. doi: 10.1111/jcmm.17233 (PMC8980952; doi:10.1111/jcmm.17233)
Supplement: Supplementary file 6 — Supplementary Material [file JCMM-26-2104-s001.docx]

***Comment 5 (Details)***

***Figure No. Figure 6C***


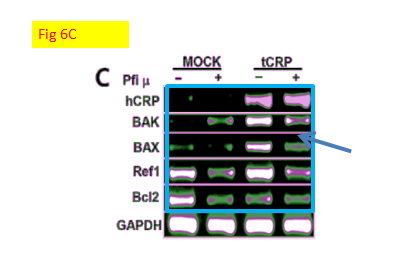


**GAPDH**

**Pfi μ**

**hCRP**


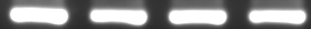

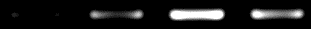


**BAK**

**BAX**


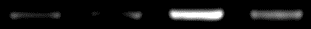


**Ref1**


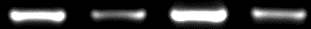


**Bcl2**


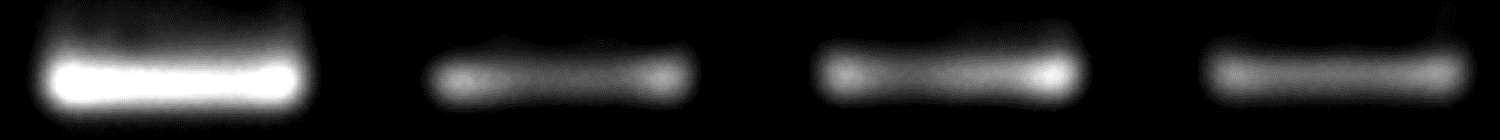

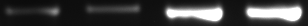


**+**

**−**

**+**

**−**

**MOCK**

**tCRP**

**C**

**Original Image**

**Analysed Image**

***Adjustments in Photoshop (brightness/ curves/ contrast) were used to analyze the image. The analysis indicates that the background of the bands in most of the lane is very clear (and not smudgy; as indicated in a blue box and an arrow), which suggests that the bands might have been placed into a standard background. Hence, the authors should be requested to provide an explanation and raw data (original gel blots) for verification.***

***Comment 5 (Author Request).***

***The authors should be requested to provide an explanation and raw data (original gel blots) for verification.***

**Response 5:**

Regarding CRP and GAPDH, there is original data in the experimental notes dated July 16, 2009. In this experiment note, 4 original bands are indicated in red text (Response 5-1). Next, regarding BAX, BAK, and Ref-1, there is original data in the experimental notes dated July 16, 2009 (Response 5-2). In Response 5-3, we marked the original bands of BAK, BAX, and Ref-1 in yellow.

Under the same conditions as in Figure 6C, the experiment was replicated twice for 16 hours and twice for 24 hours then, gel loading was performed. The experimental results for 24 hours were selected as the figure. Overall, the brightness and contrast has been adjusted to be excessive than in the original data. However, any other artificial manipulation was not performed.


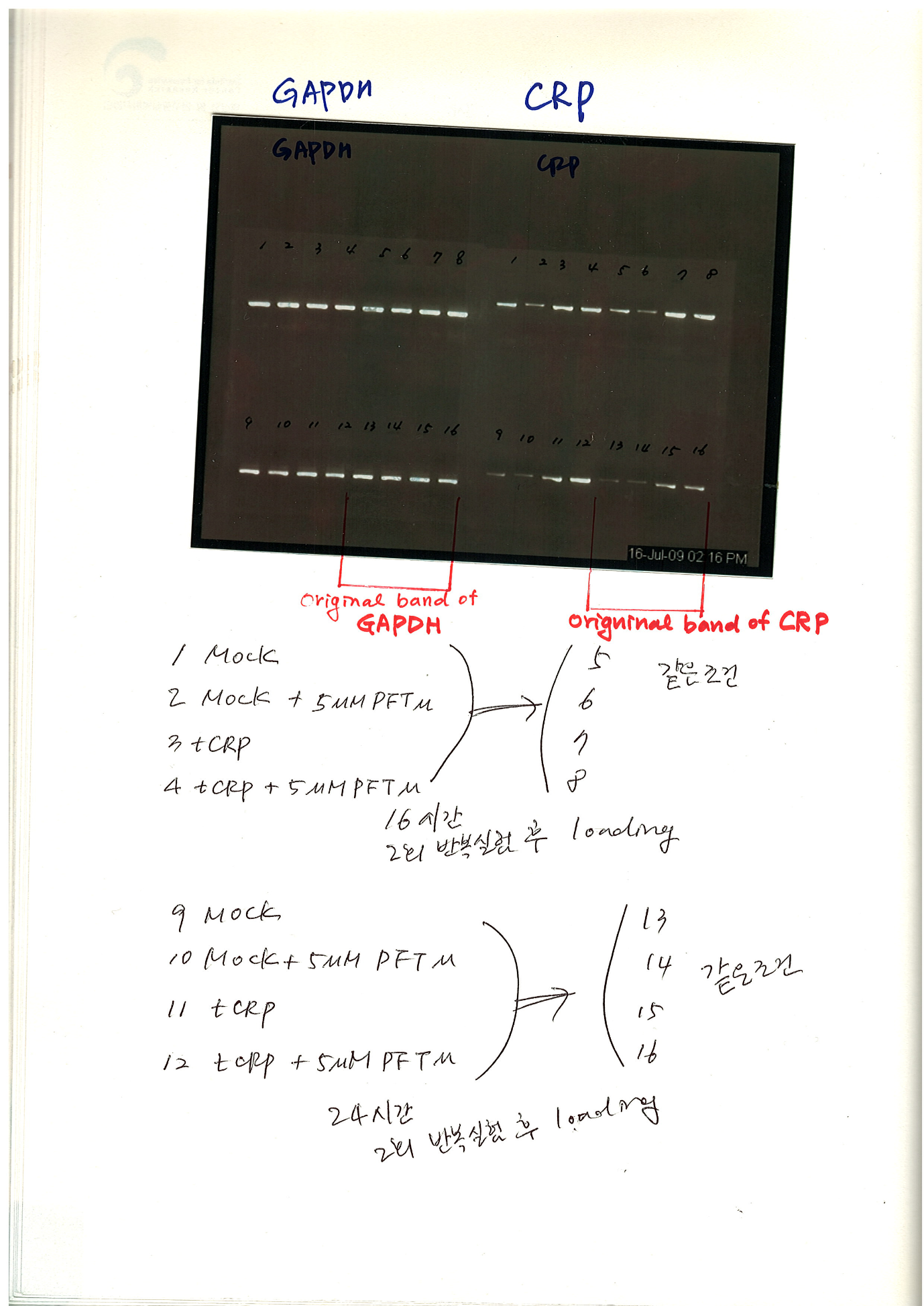


Response 5-1. Experimental note for CRP band (marked in Red Text)

***

***

Response 5-2. Experimental note for BAK, BAX, and Ref1 band

***
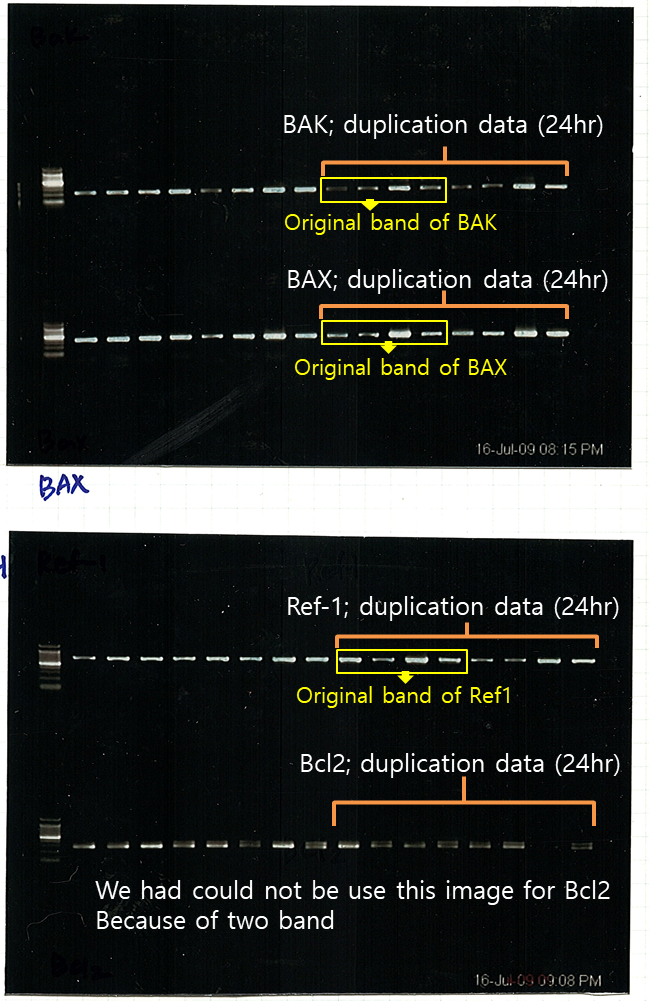
***

Response 5-3. Description for the original BAK, BAX, and Ref1 band of Figure 6A (marked in yellow)

At that time, the Bcl2 band resulted in as two bands in our experiment. we didn't use this image. After re-designing the rat Bcl2 primer, we performed the same experiment again to obtain the Bcl2 band. Unfortunately, we cannot currently find some of Bcl2 data. At the time, we shared equipment. Some of our original data could not be obtained before the equipment disposal action by the equipment manager. Currently, we only have the data adjusted the brightness and contrast.

However, we can present the other original experimental notes that confirmed the same results for Bcl2 (Response5-4). In this experimental note, the Bcl2 band arrangement is different, but it shows the same result of Bcl2 as figure 6C. This is the experimental result of re-verification of Bcl2 expression levels to PTFu drug response when CRP is overexpressed.

In the original Gel Doc images in the experimental note, the Bcl2 band arrangement as same condition of Figure 6C is indicated in yellow text (Response 5-5).

Based on the original photo images of CRP, BAK, BAX, Ref-1, Bcl2, and GAPDH, a new figure 6C (Response 5-6) was created and reflected by replacing the previous figure. We hope that you confirm and approve the figure replacement.





Response 5-4. Another experimental note for Bcl2 band

***
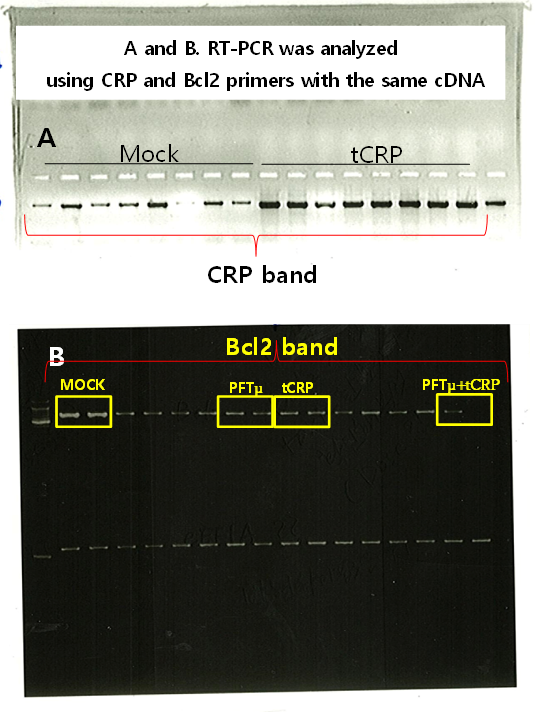
***

Response 5-5. Description of Bcl2 band of (Response 5-4)

**GAPDH**

**Pfi μ**

**hCRP**

**BAK**

**BAX**

**Ref1**

**Bcl2**

**+**

**−**

**+**

**−**

**MOCK**

**tCRP**

**Fig 6C**


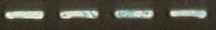

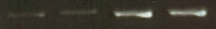

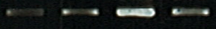

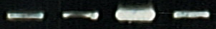

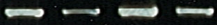

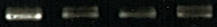


Response 5-6. The new image of Figure 6C
