# Supplementary material for: Radiation‐induced C‐reactive protein triggers apoptosis of vascular smooth muscle cells through ROS interfering with the STAT3/Ref‐1 complex
Source: J Cell Mol Med. 2022 Feb 17;26(7):2104–18. doi: 10.1111/jcmm.17233 (PMC8980952; doi:10.1111/jcmm.17233)
Supplement: Supplementary file 7 — Raw data Fig S7a [file JCMM-26-2104-s005.docx]

***Comment 6 (Details)***

***Figure No. Supplementary Figure 7A***


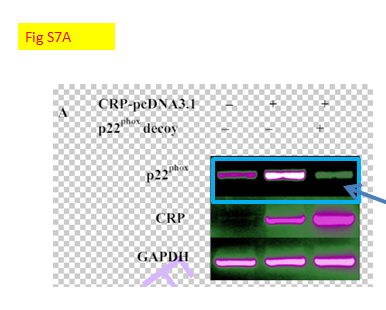

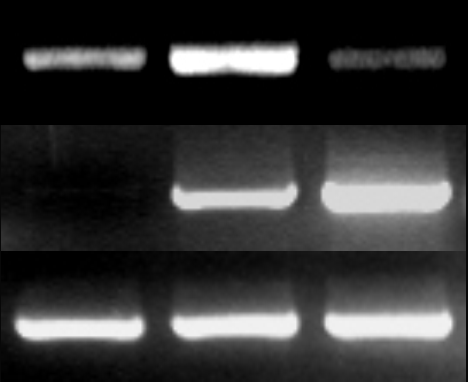


**p22^phox^**

**CRP**

**GAPDH**

**CRP-pcDNA3.1 – + +**

**p22^phox^ decoy – – +**

**A**

**Original Image**

**Analysed Image**

***Adjustments in Photoshop (brightness/ curves/ contrast) were used to analyze the image. The analysis shows that the background of the bands in 'p22' lane is very clear (and not smudgy; highlightes using a blue box and an arrow), which suggests that the bands might have been placed into a standard background. Hence, the authors should be requested to provide raw data (original gel blots) for verification.***

***Comment 6 (Author Request).***

***The authors should be requested to provide an explanation and raw data (original gel blots) for verification.***

**Response 6:**

We are presenting the original Gel Doc image of p22^phox^ in figure S7A (Response 7-1).

A detailed description of how it was modified is expressed in the figure below (Response 7-2). This is the result of confirming that p22^phox^ mRNA expression was suppressed by p22^phox^ -decoy through repeated experiments 4 times. However, the 3rd band of p22^phox^ was deleted to match the band arrangement of CRP loading.

To emphasize the viewpoint where p22^phox^ expression is suppressed by p22^phox^-decoy in CRP overexpressed VSMCs, the 4th band to be emphasized was moved to the 3rd band region.

Based on the original Gel Doc images of p22^phox^, CRP, and GAPDH, a new Supplementary figure S7A has been created and replaced in the revised Supplementary Figure (Response 6-3). We hope that you confirm and approve the figure replacement.


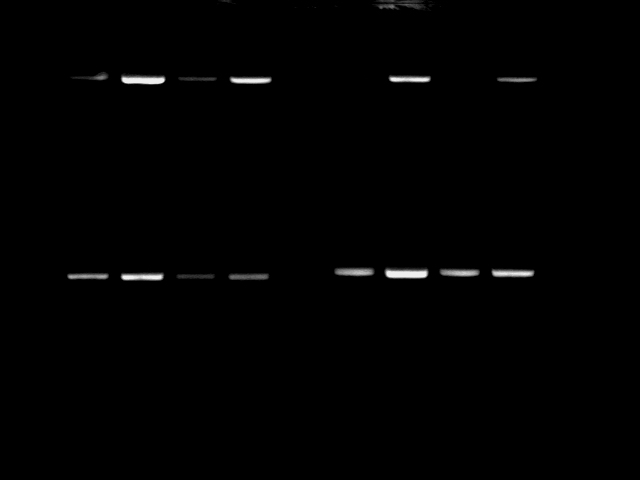


Response 6-1. Gel Doc Image of P22^phox^ band


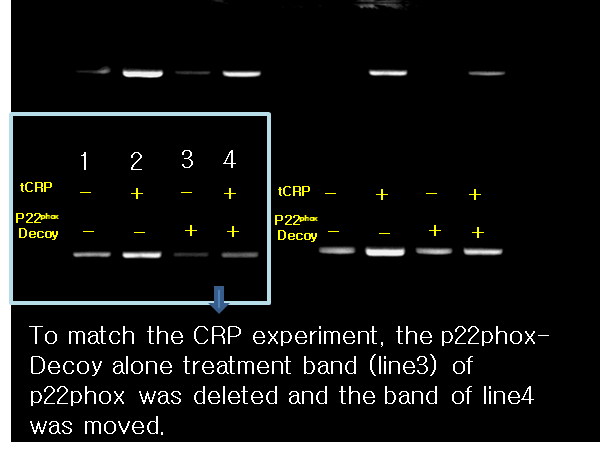


Response6-2. Description for p22^phox^ band of (Response 6-1)

**p22^phox^**

**CRP**

**GAPDH**

**CRP-pcDNA3.1 – + +**

**p22^phox^ decoy – – +**

**S7A**


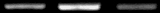

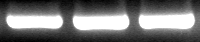

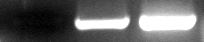


Response 6-3. The new image of Supplementary Figure S7A
