# Supplementary material for: Radiation‐induced C‐reactive protein triggers apoptosis of vascular smooth muscle cells through ROS interfering with the STAT3/Ref‐1 complex
Source: J Cell Mol Med. 2022 Feb 17;26(7):2104–18. doi: 10.1111/jcmm.17233 (PMC8980952; doi:10.1111/jcmm.17233)
Supplement: Supplementary file 8 — Raw data Fig S7a [file JCMM-26-2104-s006.docx]

***Comment 7 (Details)***

***Figure No. Supplementary Figure 7A***


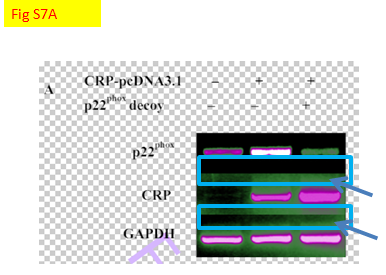

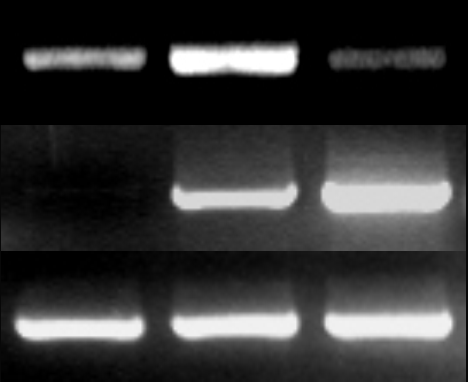


**p22^phox^**

**CRP**

**GAPDH**

**CRP-pcDNA3.1 – + +**

**p22^phox^ decoy – – +**

**A**

**Original Image**

**Analysed Image**

***Adjustments in Photoshop (brightness/curves, and contrast) were used to analyze the image. The analysis shows the presence of sharp vertical lines between the gel blocks (as highlighted in blue boxes and arrows), which indicates that three images have been combined. To present the results more transparently, the authors should be requested to add clear space or dividing lines between the gel blocks, so that it is clear to the readers that the figure is a composite of separate images.***

***Comment 7 (Author Request).***

***To present the results more transparently, the authors should be requested to add clear space or dividing lines between the gel blocks, so that it is clear to the readers that the figure is a composite of separate images.***

**Response 7:**.

In response to your request, we are presenting a new figure in the revised supplementary figure with relevant content. Based on the original Gel Doc images of p22^phox^, CRP, and GAPDH, the new figure (Response 7-1) was created. Individual gene mRNA expression levels were made into separate gel blocks and clearly separated from each other.

We hope that you confirm and approve the figure replacement.

**p22^phox^**

**CRP**

**GAPDH**

**CRP-pcDNA3.1 – + +**

**p22^phox^ decoy – – +**

**S7A**


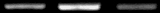

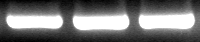

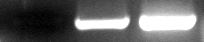


Response 7-1. New image of Supplementary Figure S7A.
